# Similar Piperacillin/Tazobactam Target Attainment in Obese versus Nonobese Patients despite Differences in Interstitial Tissue Fluid Pharmacokinetics

**DOI:** 10.3390/pharmaceutics13091380

**Published:** 2021-08-31

**Authors:** David Busse, Philipp Simon, David Petroff, Christoph Dorn, Lisa Schmitt, Davide Bindellini, Alexander Kratzer, Arne Dietrich, Markus Zeitlinger, Wilhelm Huisinga, Robin Michelet, Hermann Wrigge, Charlotte Kloft

**Affiliations:** 1 Department of Clinical Pharmacy and Biochemistry, Institute of Pharmacy, Freie Universitaet Berlin, 12169 Berlin, Germany; david.busse@fu-berlin.de (D.B.); lisa.ehmann@fu-berlin.de (L.S.); davide.bindellini@fu-berlin.de (D.B.); robin.michelet@fu-berlin.de (R.M.); 2 Graduate Research Training Program PharMetrX, 12169 Berlin, Germany; 3 Department of Anesthesiology, Intensive Care, University of Leipzig Medical Centre, 04103 Leipzig, Germany; philipp.simon@medizin.uni-leipzig.de (P.S.); arne.dietrich@medizin.uni-leipzig.de (A.D.); 4 Integrated Research and Treatment Center (IFB) Adiposity Diseases, University of Leipzig, 04103 Leipzig, Germany; david.petroff@zks.uni-leipzig.de (D.P.); hermann.wrigge@bergmannstrost.de (H.W.); 5 Clinical Trial Centre Leipzig, University of Leipzig, 04109 Leipzig, Germany; 6 Institute of Pharmacy, University of Regensburg, 93053 Regensburg, Germany; Christoph.Dorn@ur.de; 7 Hospital Pharmacy, University Hospital Regensburg, 93053 Regensburg, Germany; Alexander.kratzer@ukr.de; 8 Department of Clinical Pharmacology, University Medical University of Vienna, 1090 Vienna, Austria; markus.zeitlinger@meduniwien.ac.at; 9 Institute of Mathematics, University of Potsdam, 14469 Potsdam, Germany; huisinga@uni-potsdam.de; 10 Department of Anesthesiology, Intensive Care and Emergency Medicine, Pain Therapy, Bergmannstrost Hospital Halle, 06112 Halle, Germany

**Keywords:** piperacillin/tazobactam, target-site, obesity

## Abstract

Precision dosing of piperacillin/tazobactam in obese patients is compromised by sparse information on target-site exposure. We aimed to evaluate the appropriateness of current and alternative piperacillin/tazobactam dosages in obese and nonobese patients. Based on a prospective, controlled clinical trial in 30 surgery patients (15 obese/15 nonobese; 0.5-h infusion of 4 g/0.5 g piperacillin/tazobactam), piperacillin pharmacokinetics were characterized in plasma and at target-site (interstitial fluid of subcutaneous adipose tissue) via population analysis. Thereafter, multiple 3–4-times daily piperacillin/tazobactam short-term/prolonged (recommended by EUCAST) and continuous infusions were evaluated by simulation. Adequacy of therapy was assessed by probability of pharmacokinetic/pharmacodynamic target-attainment (PTA ≥ 90%) based on time unbound piperacillin concentrations exceed the minimum inhibitory concentration (MIC) during 24 h (%*f*T_>MIC_). Lower piperacillin target-site maximum concentrations in obese versus nonobese patients were explained by the impact of lean (approximately two thirds) and fat body mass (approximately one third) on volume of distribution. Simulated steady-state concentrations were 1.43-times, 95%CI = (1.27; 1.61), higher in plasma versus target-site, supporting targets of %*f*T**_>2×MIC_** instead of %*f*T**_>4×MIC_** during continuous infusion to avoid target-site concentrations constantly below MIC. In all obesity and renally impairment/hyperfiltration stages, at MIC = 16 mg/L, adequate PTA required prolonged (thrice-daily 4 g/0.5 g over 3.0 h at %*f*T_>MIC_ = 50) or continuous infusions (24 g/3 g over 24 h following loading dose at %*f*T_>MIC_ = 98) of piperacillin/tazobactam.

## 1. Introduction

In recent decades, obesity has increased in prevalence globally (mean prevalence = 19.5% in OECD countries in 2015 [1]). Alarmingly, in the United States, over 40% of adults are obese [2] with projections of up to 50% in 2030 [3]. This challenges health care systems, e.g., with a higher risk of mortality from serious bacterial infections, including surgical site infections in critically ill, obese compared with nonobese patients [4,5,6,7]. The optimization of antimicrobial dosing regimens is crucial to maximize the treatment success in these patients. Yet, only sparse data are available on the impact of obesity-related changes of antimicrobial pharmacokinetics (PK) [8,9,10], hampering the development of population PK models and their use in model-informed precision dosing to guide antibiotic dosing regimens [11,12]. Previous investigations on the effect of body mass on the PK of piperacillin, a β-lactam antibiotic with bactericidal activity against a broad spectrum of Gram-negative and Gram-positive bacteria [13], have been compromised by inconsistent scaling approaches of PK parameters with body mass [8,14]. This has hindered their implementation in model-informed personalization of antimicrobial dosing.

Piperacillin is commonly co-administered with the β-lactamase inhibitor tazobactam to enhance its activity against β-lactamase-producing pathogens [15]. The bactericidal activity of piperacillin is time-dependent, and the PK/pharmacodynamic (PD) parameter best predicting clinical and microbiological outcomes is the fraction of time that unbound (i.e., “free”) piperacillin concentrations exceed the minimum inhibitory concentration (MIC) during the dosing interval (%*f*T_>MIC_) [16]. For piperacillin, %*f*T_>MIC_ ≥ 50 is considered necessary for optimal activity [17] and %*f*T_>MIC_ ≥ 100 has been recommended for infections in critically ill patients [18]. For β-lactams, continuous infusions have been recommended [19,20,21] with PK/PD targets related to multiples of the MIC (i.e., %*f*T_>**4×MIC**_ ≥ 50 or 100) to avoid concentrations at the site of infection (target-site) below MIC for the entire dosing interval [22,23].

Yet, piperacillin exposure at the target-site in (morbidly) obese patients, measured by the gold standard microdialysis, a minimally invasive technique to measure unbound drug concentrations at the target-site [24], has never been quantified. Such data are still limited to few antibiotics [25,26,27] and are urgently needed to investigate if adaptations of dosing regimens in (morbidly) obese patients are necessary. For this purpose, the European Medicines Agency recommended probability of target attainment (PTA) analysis [24]. PTA is based on plasma data but it is unknown whether presumably effective piperacillin exposure in plasma (with targets based on clinical data in nonobese patients) translates to effective exposure at the target-site of obese patients. Therefore, authors of recent publications on piperacillin PK in obese individuals have suggested investigating effective piperacillin concentrations at the target-site [14,28,29,30].

The aim of this analysis was to assess appropriateness and potentially adapt the currently used piperacillin/tazobactam dosing regimens in obese and nonobese patients. For this, the most adequate body size descriptor and further influential patient factors to adjust piperacillin/tazobactam dosing regimens were identified and target-site penetration in obese and nonobese patients was assessed.

## 2. Materials and Methods

### 2.1. Study Design and Patient Population

Data from a prospective, parallel-group, open-label, controlled single-center trial (EudraCT No. 2012-004383-22) were analyzed. Detailed information concerning the study design, procedures, and data collection has been described elsewhere [31]. Inclusion criteria were: abdominal surgery, age ≥ 18 years, BMI = 18.5–30 kg/m^2^ for nonobese and BMI ≥ 35 kg/m^2^ for obese patients. By design, patients with BMI 30.0 kg/m^2^ to 34.9 kg/m^2^ were not included to have clearer separation between the patient groups. Nonobese patients were age- and sex-matched to the obese patient group.

Patients received a standard (weight-independent) single i.v. infusion of 4 g/0.5 g piperacillin/tazobactam through an additional venous access over 30 min. Dense blood sampling (pre-dose and after 0.5,1, 2, 3, 4, 5, 6, and 8 h) and collection of microdialysate samples in interstitial space fluid (ISF) of subcutaneous adipose tissue (pre-dose and 0–0.5, 0.5–1, 1–1.5, 1.5–2, 2–3, 3–4, 4–5, 5–6, 6–7, and 7–8 h) of both upper arms (one catheter per arm to quantify microdialysis method-related variability [32,33]) were performed as described in [31]. To derive drug concentration in ISF from microdialysate concentrations, the retrodialysis calibration method was used [34].

### 2.2. Bioanalysis of Piperacillin Concentrations

Piperacillin concentrations were determined by high-performance liquid chromatography with photometric detection. The analytical column was a XBridge C18 BEH 2.5 µ, 50 × 3 mm (Waters, Eschborn, Germany). The mobile phase consisted of 0.05 M NaH_2_PO_4_/MeCN 83:17 (*v*/*v*), pH 6.4. The flow rate was 0.4 mL/min.

Unbound plasma piperacillin concentrations were determined after ultrafiltration. Based on in-process quality controls the coefficient of variation (CV) of intra-/inter-assay precision of the determination of total drug in plasma was <4% <6%, the accuracy was 101%. The intra-assay precision of the determination of unbound drug in plasma was not further examined, as in preliminary experiments the difference between samples analyzed in duplicate was as low as 1%, i.e., in the range of the precision of the injection system. The inter-assay precision was assessed by analyzing spiked plasma of healthy subjects with total piperacillin concentrations between 1 and 100 mg/L. The unbound fraction in these samples was 85.7 ± 3.2% (CV 3.7%).

The CV of the intra-/inter-assay precision in 0.9% NaCl as surrogate for microdialysate and ultrafiltrate was <3% <8%, the accuracy was 98.5%. The stability of the processed samples in the autosampler was 99.6 ± 3.3% (total concentrations), 95.6 ± 2.4% (unbound concentrations), or 98.9 ± 4.3% (microdialysate).

The lower limit of quantification was 0.3 mg/L in plasma and 0.03 mg/L in 0.9% NaCl as surrogate for microdialysate and ultrafiltrate. Further details on the bioanalysis of piperacillin concentrations are described in [35].

### 2.3. Population Pharmacokinetic Model and Identification of Body Size Descriptors

A population PK analysis was performed to characterize the plasma and target-site PK of piperacillin simultaneously, to explain the variability in the PK parameters by patient-specific covariates and to identify which body size descriptors were most predictive of piperacillin PK [36]. The 2–3-compartment PK models with plasma data attributed to the central and target-site data to the central or peripheral compartment were evaluated using the integrated dialysate-based modeling approach, as described in detail in [37,38]. 

Clinical and demographic characteristics (body size descriptors, predicted glomerular filtration rate and creatinine clearance), which were considered biologically plausible to affect piperacillin PK, were tested for inclusion as covariates. Statistically significant differences (*p* < 0.05) in individual parameter estimates between obese and nonobese patients were investigated by Mann–Whitney–Wilcoxon tests. Population PK models were evaluated by standard goodness-of-fit plots and the predictive model performance was assessed by visual predictive checks (*n* = 1000) [39]. Further information on population PK model development is provided in the Supplementary Section “Model development”.

### 2.4. Target-Site Penetration in Obese and Nonobese Patients

To evaluate the impact of obesity stages on predicted exposure in plasma and at the target-site and to quantify target-site penetration, two virtual reference patients were defined (Table 1):

(1) Nonobese, healthy renal function patient
(2) Morbidly obese, healthy renal function patient

Reference patient (1) was a male individual with standard total body weight = 70.0 kg, standard body height = 1.76 m [44] (BMI = 22.6 kg/m^2^) and healthy renal function (eGFR = 90.0 mL/min/1.73 m^2^ [42]). Reference patient (2) was defined by the lower BMI threshold for morbid obesity (BMI = 40.0 kg/m^2^ [45], standard body height = 1.76 m, total body weight = 127 kg). Since the body surface area is substantially higher in obese compared to nonobese individuals [46] (compare Table 1), eGFR related to healthy renal function for patient (2) was adjusted by “de-indexing” [47] 90.0 mL/min/1.73 m^2^: The employed calculated body surface area [41] of reference patient (2) resulted in eGFR = 130 mL/min (Table 1). The body size descriptor lean body weight (LBW) was derived from the patient information via [40] and fat mass was calculated as the difference between individual total body weight and LBW.

To evaluate the effect of renal function on piperacillin exposure and target-site penetration, two additional reference patients were defined (Table 1):

(3) Nonobese, renally impaired patient
(4) Nonobese, hyperfiltration patient

Reference patient (3) was defined by the lower threshold of CKD stage 3A (creatinine clearance calculated via Cockcroft–Gault based on adjusted body weight, CLCR_CG_ABW_ = 45.0 mL/min, minimum observed in present study CLCR_CG_ABW_ = 49.8 mL/min) and the nonobese, hyperfiltration (4) reference patient was defined by the lower threshold of hyperfiltration (CLCR_CG_ABW_ = 130 mL/min [48]).

Simulations of unbound piperacillin plasma and target-site concentrations over 8 h following a short-term infusion of 4 g/0.5 g piperacillin/tazobactam administration were performed and median differences in minimum and maximum piperacillin concentrations between reference patients were calculated.

To account for the time-dependent antibiotic effect of piperacillin, the target-site:plasma ratio of %*f*T_>MIC_ was investigated (effective penetration index). The following MIC values were selected: MIC = 2 mg/L (epidemiologic cutoff value (ECOFF) of *Staphylococcus aureus*), 4 mg/L, 8 mg/L (Gram-positive and Gram-negative anaerobes and species independent susceptibility breakpoint; ECOFF of *Escherichia coli*, *Klebsiella pneumoniae*) and 16 mg/L (Gram-positive and Gram-negative anaerobes and species independent resistance breakpoint, ECOFF of *Pseudomonas aeruginosa*) [49].

To investigate if PK/PD targets related to 4×MIC (i.e., %*f*T_>**4×MIC**_) for continuous infusions are suitable to avoid target-site concentrations below MIC for the entire dosing interval and for all four reference patients (1)–(4), the steady-state plasma:target-site unbound concentration ratio was evaluated.

### 2.5. Evaluation of Clinically Relevant Piperacillin/Tazobactam Dosing Regimens

To evaluate the impact of obesity status and renal function on PTA, the four reference patients (1)–(4) were selected for calculation of PTA (Monte-Carlo simulation; *n* = 1000 per reference patient) for the species-independent resistance breakpoint (MIC = 16 mg/L). To systematically evaluate the impact of body mass and renal function on the PTA of piperacillin/tazobactam, Monte-Carlo simulations were performed for the identified model covariates with the observed ranges in this clinical study (*n* = 1000 per covariate combination) using the developed population PK model.

The PK/PD index %*f*T_>MIC_ was evaluated for the PK/PD targets %*f*T_>MIC_ = 50 (optimal activity [17]) and %*f*T_>MIC_ = 98. The latter was selected instead of %*f*T_>MIC_ = 100 (critically ill patients [18]), since treatment at day 1 was evaluated during PTA analysis and piperacillin concentrations in all matrices are zero before start of the first dose, preventing any concentration-time profile from attaining %*f*T_>MIC_ = 100. For continuous infusions, a stricter PK/PD target of %*f*T_>**4×MIC**_ [22] was selected. MIC = 2.00–16.0 mg/L values for piperacillin/tazobactam were evaluated.

To determine if piperacillin/tazobactam dosing regimens achieve effective concentrations in obese and nonobese patients in clinical routine, the sum of PTA weighted by the relative frequency of MIC values (Supplementary Figure S1), the “cumulative fraction of response” (CFR [50]), for specific populations of microorganisms was calculated. Infections by pathogens commonly treated with piperacillin/tazobactam were selected (*Escherichia coli*, *Klebsiella pneumoniae*, *Pseudomonas aeruginosa*, *Streptococcus pneumoniae,* and *Staphylococcus aureus*). A dosing regimen was considered adequate if PTA was ≥90% [24]; the same threshold was selected for CFR [51,52]. Four dosing regimens were evaluated in the PTA and CFR analysis (Table 2).

## 3. Results

### 3.1. Patient Population

Thirty patients (15 obese and 15 nonobese) scheduled for elective abdominal surgery were recruited according to study protocol [31]. The cohort comprised male (12/30 patients) and female patients, covered a wide body mass range (BMI_nonobese_ = 20.1–29.3 kg/m^2^ and BMI_obese_ = 37.5–52.0 kg/m^2^, Table 3) and a wide range of renal functions from moderately impaired to augmented renal function (CLCR_CG_ABW_ = 49.8–173 mL/min, Supplementary Figure S2) with none of the patients requiring haemodialysis. As expected, both lean body weight (LBW, Supplementary Figure S3) and fat mass were higher in obese compared to nonobese patients (Table 3).

In total, 1069/1128 (observed/planned) piperacillin concentrations were available, comprising total (*n* = 237/240) and unbound (*n* = 114/116) plasma concentrations, microdialysate concentrations collected via two catheters in the ISF of subcutaneous adipose tissue over 8 h (*n* = 285/300 + 272/300; Supplementary Figure S4), and retrodialysate concentrations (*n* = 80/86 + 81/86).

### 3.2. Population Pharmacokinetic Model and Identification of Body Size Descriptors

**Figure 1:**
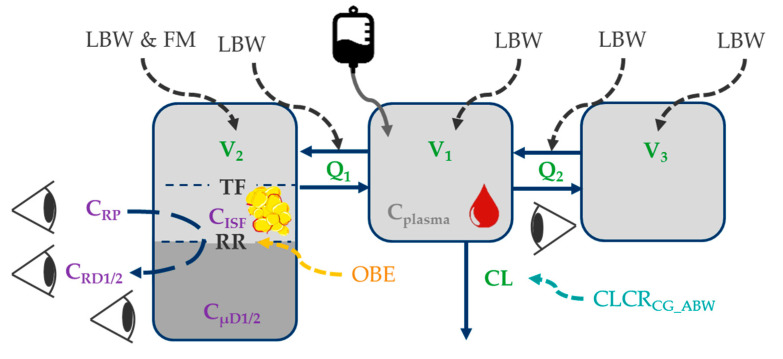
Illustration of the final piperacillin population pharmacokinetic model. Impact of patient characteristics on structural pharmacokinetic parameters (green); black font: body size descriptors; orange font: categorical difference between obese and nonobese patients; turquoise font: renal function); purple font: microdialysis-related observation types; Eyes: observations in plasma, plasma after ultrafiltration, microdialysate, retrodialysate. Abbreviations: LBW: Lean body weight; FM: Fat mass; CLCR_CG_ABW_: Creatinine clearance calculated via Cockcroft-Gault based on adjusted body weight; C_ISF_: Piperacillin concentration in interstitial space fluid of subcutaneous adipose tissue; C_plasma_: Total plasma concentration; C_RD1/2_: Retrodialysate concentration from catheter 1/2; C_RP_: Retroperfusate concentration; C_µD1/2_: Microdialysate concentration from catheter 1/2; Q_1_/Q_2_: Intercompartmental flows; RR: Relative recovery; TF: Tissue factor relating predicted concentrations of “shallow” compartment to observed C_ISF_; V_1_/V_2_/V_3_: Volume of distribution parameters of central/peripheral compartments.

Observed concentrations in all matrices (plasma, plasma after ultrafiltration, microdialysate, retrodialysate) were best characterized by a three-compartment model (Figure 1; Supplementary Table S1). Target-site concentrations were related to a peripheral compartment, which was best described by a tissue factor of 69.9% 95%CI = (62.3%; 75.4%) (Supplementary Table S2), scaling predicted piperacillin concentrations in this peripheral compartment to target-site concentrations (Figure 1).

The individual total volumes of distribution (V_1_ + V_2_ + V_3_, median (range)) were similar in obese (25.0 L (14.9–56.6 L)) and nonobese patients (22.1 L (13.7–48.4 L), *p* = 0.486). However, higher individual volumes of distribution associated with the target-site (V_2_, Figure 1) were evident in obese (4.64 L (2.54–7.16 L)) versus nonobese patients (2.50 L (1.94–4.70 L), *p* < 0.001). Individual clearance values were similar in obese (17.4 L/h (9.34–31.2 L/h)) and nonobese patients (16.8 L/h (9.86–37.0 L/h), *p* = 0.367).

Given the similarity of observed maximum piperacillin concentrations in plasma in obese and nonobese patients (Supplementary Figure S5, left and mid), allometric scaling of the central (V_1_) and the “deep” (i.e., *slow* mass transfer from central to this peripheral compartment) peripheral volume (V_3_) via LBW (not total body weight) was most adequate (Supplementary Table S1). Similarly, the intercompartmental flows (Q_1_ and Q_2_) were scaled based on LBW [53].

In contrast, a physiologically-motivated impact of LBW [40] and fat mass (FM = total body weight − LBW) on V_2_ was identified (Supplementary Table S2 and [54]), demonstrating that the impact of LBW (68.4%) was higher than that of fat body mass (31.6%). V_2_ was associated with the “shallow” peripheral compartment (i.e., *fast* mass transfer from central to this peripheral compartment), which was associated with observed target-site concentrations (Figure 1). 

Piperacillin clearance increased by 0.583 L/h per 10 mL/min increase of CLCR_CG_ABW_ (Supplementary Table S2). No additional impact of LBW on piperacillin clearance was identified (Supplementary Table S1).

Implementation of these covariate effects resulted in an adequate description of the observed trends of individual CL with CLCR_CG_ABW_ (Supplementary Figure S6B) and volumes of distribution with LBW and fat mass (Supplementary Figure S6C–F), as judged by inspection of individual random-effects parameter values versus these covariates. 

All piperacillin model parameters were sufficiently precisely estimated (RSE ≤ 41.0%; Supplementary Table S2), the results of model evaluation demonstrated appropriate model performance for all four evaluated matrices (total plasma, unbound plasma, microdialysate, and retrodialysate; Supplementary Figure S7) and adequate model predictive performance was shown by visual predictive check (Supplementary Figure S8).

The retroperfusate concentration for both microdialysis catheters was missing in one obese patient and was imputed by the nominal concentration. A sensitivity analysis to investigate the impact of different imputations on PK parameter estimates proved a negligible impact of the choice of imputation strategy (Supplementary Figure S9).

### 3.3. Target-Site Penetration in Obese and Nonobese Patients

The impact of obesity status and renal function on target-site penetration was evaluated based on the “standard dosage 1” of piperacillin (Supplementary Figure S10). The median effective penetration index (target-site:plasma ratio of %*f*T_>MIC_) was similar in all reference patients for each investigated MIC (Supplementary Figure S11C,G). A detailed description on the impact of obesity status and renal function on unbound piperacillin exposure in plasma and at target-site is presented in the Supplementary Section “Target-site penetration in obese and nonobese patients”.

To investigate if PK/PD targets related to 4×MIC (%*f*T_>**4×MIC**_) for continuous infusions preceded by a 0.5 h loading dose are suitable to avoid target-site concentrations below MIC for the entire dosing interval for all reference patients (1)–(4), the simulated plasma and target-site concentrations following this “continuous infusion” dosing regimen were evaluated: Unbound piperacillin steady-state concentrations were 1.43-times, 95%CI = (1.27; 1.61), higher in plasma versus target-site for all reference patients (Supplementary Figure S12).

### 3.4. Evaluation of Clinically Relevant Piperacillin/Tazobactam Dosing Regimens

**Figure 2:**
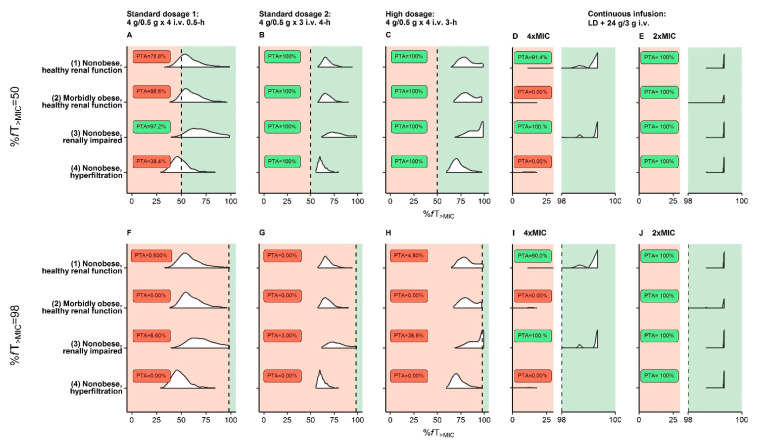
Probability of target attainment (PTA) based on the EUCAST species-independent resistance breakpoint (MIC = 16 mg/L) for virtual reference patients (see Table 1) receiving three dosing regimens recommended by EUCAST (**A**/**F**,**B**/**G**,**C**/**H**) and one continuous infusion dosing regimen (**D**/**E**,**I**/**J**). (**D**/**E**,**I**/**J**): Pharmacokinetic/pharmacodynamic targets related to 4×MIC or 2×MIC for the intervals %*f*T_>MIC_ = 0–30 and %*f*T_>MIC_ = 98–100 to allow discrimination between PTA curves <30% and >98%; vertical dashed black line: pharmacokinetic/pharmacodynamic targets (%*f*T_>MIC_ = 50 or %*f*T_>MIC_ = 98); PTA ≥ 90% (adequate therapy) labeled as green area and PTA < 90% (inadequate therapy) as red area. Abbreviations: %*f*T_>MIC_: Fraction of time that unbound concentrations exceed MIC over 24 h; EUCAST: European Committee on Antimicrobial Testing; i.v.: Intravenous; MIC: Minimum inhibitory concentration.

For the species-independent resistance breakpoint of piperacillin/tazobactam (MIC = 16.0 mg/L) and the PK/PD target %*f*T_>MIC_ = 50, adequate PTA was achieved for all reference patients (1)–(4) by prolonged infusions of piperacillin/tazobactam (Figure 2B,C).

For the same breakpoint and a stricter PK/PD target (%*f*T_>MIC_ = 98), PTA was only adequate for the nonobese, healthy renal function (1) and nonobese, renally impaired reference patient (3) and only for the dosing regimen “continuous infusion” (Figure 2I). Applying a more lenient PK/PD target related to 2×MIC in continuous infusion, which still conservatively accounted for the lower plasma:target-site steady-state concentration (Supplementary Figure S12), PTA = 100% was reached in all reference patients (1)–(4) (Figure 2J).

When evaluating PTA over the entire study range of body mass and CLCR_CG_ABW_ and for MIC = 2.00–16.0 mg/L and %*f*T_>MIC_ = 50 only the “high dosage” prolonged infusions were adequate for all patients and even when applying this PK/PD target to target-site exposure (Supplementary Figure S13E,F). By applying the PK/PD target %*f*T_>MIC_ = 98 to target-site exposure (related to 1×MIC), it was demonstrated that only the dosing regimen “continuous infusion” was adequate for all patients (Supplementary Figure S14H; including patients with CLCR_CG_ABW_ = 170 mL/min).

**Figure 3:**
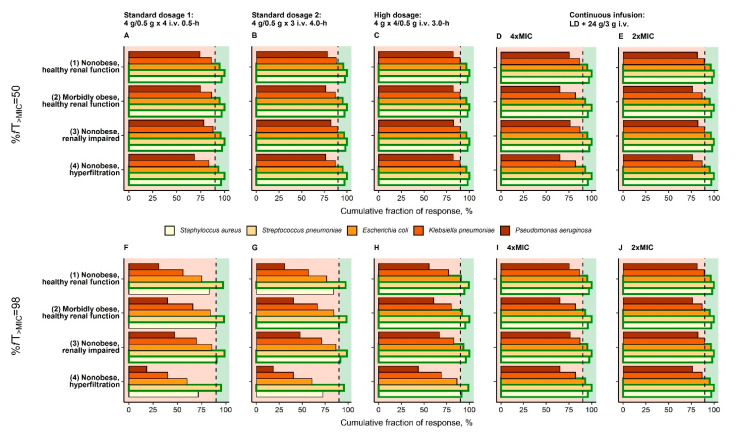
Cumulative fraction of response for virtual reference patients (see Table 1) receiving three dosing regimens recommended by EUCAST (**A**/**F**,**B**/**G**,**C**/**H**) and one continuous infusion dosing regimen (**D**/**E**,**I**/**J**) based on the EUCAST species-specific distributions of MIC. Vertical dashed black line: Separation of CFR < 90% (inadequate therapy) and CFR ≥ 90% (adequate therapy); Bars bordered by green line: CFR ≥ 90% for bacterial species. Abbreviations: %*f*T_>MIC_: Fraction of time that unbound concentrations exceed MIC over 24 h; CFR: Cumulative fraction of response; EUCAST: European Committee on Antimicrobial Testing; i.v.: Intravenous; MIC: Minimum inhibitory concentration.

For %*f*T_>MIC_ = 50, CFR of *S. aureus*, *S. pneumoniae*, *E. coli*, and *K. pneumoniae* was adequate for the prolonged “high dosage” for all reference patients (Figure 3C), whereas for *P. aeruginosa* CFR was inadequate for all investigated dosing regimens. For %*f*T_>MIC_ = 98 the dosing regimen “continuous infusion” reached adequate CFR for all reference patients and *S. aureus*, *S. pneumoniae* and *E. coli*, but none of the investigated dosing regimens was adequate for *P. aeruginosa* and *K. pneumoniae*, because of their high relative frequencies at high MIC values (MIC > 16 mg/L; Supplementary Figure S1).

## 4. Discussion

For infections with MIC = 2–16 mg/L and applying a lenient PK/PD target (%*f*T_>MIC_ = 50) prolonged “high dosage” i.v. piperacillin/tazobactam (four-times daily 4 g/0.5 g over 3.0 h) might suffice to treat patients of all categories of obesity and renal function, even when applying the same PK/PD targets to the target-site. For the stricter PK/PD target (%*f*T_>MIC_ = 98) and MIC = 16 mg/L, neither the intermittent dosing regimens nor the continuous infusion dosing regimen (%*f*T**_>4×MIC_** = 98) were adequate in any investigated category of obesity and renal function. Yet, the commonly employed 4×MIC in continuous infusion proved too conservative given only 1.43-times, 95%CI = (1.27; 1.61) higher unbound piperacillin concentrations in plasma versus target-site. Relating PK/PD targets to 2×MIC was therefore deemed sufficiently cautious to evaluate PTA of continuous infusions in patients of all obesity classes: When applying %*f*T**_>2×MIC_** = 98 to the continuous infusion dosing regimen and MIC = 16 mg/L, a continuous i.v. infusion of 24 g/3 g piperacillin/tazobactam over 24 h following a 4 g/0.5 g i.v. short-term loading dose proved adequate in all categories of obesity and renal function.

These PK/PD evaluations of dosing regimens were based on the relationships of (i) renal function and (i) body size descriptors with piperacillin PK parameters, which have been quantified in this analysis based on a population PK model. The identified impact of renal function on piperacillin CL was consistent with previous studies [14,28]. However, the lack of a standardized estimation of CLCR_CG_ in obese patients has been recognized as a major challenge in dosing of antibiotics [55]. The least biased body size descriptor in the calculation of CLCR_CG_ over a large BMI range was reported to be adjusted body weight [56], although in the present study other estimators of renal function resulted in equally accurate characterizations of the presented piperacillin exposure data. The larger CLCR_CG_ABW_ in obese patients obtained in our analysis is supported by the reported initial state of glomerular hyperfiltration in obesity [57]. Neither an additional impact of body mass on piperacillin CL, as shown in [14], nor an impact of CLCR_CG_ABW_ on piperacillin fraction unbound, as reported for other drugs [58], could be identified.

The quantitative influence of the body size descriptors LBW and fat mass on piperacillin PK parameters was shown, corroborating the effect of body mass found in other analyses [14,28]: Scaling the central and one peripheral compartment (representing the vascular system and highly-perfused organs) with LBW alone and the other peripheral compartment (representing other organs such as adipose tissue) with LBW and fat mass represents a physiology-motivated distribution model [54,59]. This scaling approach allowed, for the first time, a description of the exposure differences at target-site between obese and nonobese patients and revealed that LBW (approximately two thirds of peripheral volume scaled via LBW) is more relevant for piperacillin target-site exposure predictions than fat mass (approximately one third of peripheral volume scaled via fat mass). By the improved characterization of the observed piperacillin concentrations over a wide range of LBW and fat mass and the plausible physiological interpretation, this allometric scaling approach offered clear advantages over hitherto described empirical scaling approaches of piperacillin PK [14,28]. Ultimately, the presented PK model might therefore serve as a basis for the precision dosing of piperacillin/tazobactam in the special patient population of (morbidly) obese patients.

These relationships of PK parameters with LBW and fat mass had a positive impact to achieve adequate PTA of piperacillin/tazobactam dosing regimens in obese patients: Although the unbound maximum piperacillin concentration was lower in (morbidly) obese patients due to high volumes of distribution, unbound minimum piperacillin concentrations were higher, resulting in higher PTA and CFR. Hence, obesity did not represent a risk factor for PK/PD target non-attainment as similarly concluded by others based on plasma data [14,28,29,60,61]. We could further show that prolonged and continuous infusions might be necessary for MIC ≥ 16 mg/L in obese and nonobese patients [28]. This was also supported by quantifying PTA related to the target-site for the first time. On top of the impact of body mass on PK, renal function played a dominant role in dosing regimen adjustment of piperacillin/tazobactam [21,62].

Besides evaluating piperacillin/tazobactam dosing regimens regarding target-site concentrations for the first time in obese individuals, the strengths of this study were the high-quality, rich sampling data obtained prospectively under clinical trial conditions and the inclusion of the matched nonobese control group. Notably, piperacillin PK was investigated following single-dose administration as perioperative antibiotic prophylaxis and extrapolation of the results to other relevant patient populations such as critically ill patients should be applied cautiously. Based on very large PK variability in critically ill patients, therapeutic drug monitoring for optimizations of dosing regimens might still be required [63]. Future clinical trials should include individual measurements of glomerular filtration via standards such as ^51^Cr-EDTA or measurement of creatinine concentrations in urine. This would allow a more accurate characterization of the distinct elimination processes of piperacillin. Yet, our analysis represents an important basis for future clinical target-site based PK/PD investigations of piperacillin/tazobactam in critically ill (morbidly) obese and nonobese patients, to allow the future use of model-informed precision dosing in these populations.

In conclusion, our findings suggest that the use of continuous piperacillin/tazobactam infusions (24 g over 24 h after a loading dose) might be adequate over all obesity categories for a strict PK/PD target (%*f*T_>MIC_ = 98) by demonstrating adequate effective target-site exposure. For a lenient PK/PD (%*f*T_>MIC_ = 50), four-times daily prolonged infusions (4 g over 3.0 h) were adequate for all stages of obesity and renal function.

## Figures and Tables

**Table 1:** Virtual reference patients of the same standard body height (1.76 m) and sex (male) but different obesity stage and renal function.

Reference Patients	BMI [kg/m^2^]	Total Body Weight [kg]	Lean Body Weight [kg] ^a^	Fat Mass [kg] ^b^	%Fat Mass ^c^	BSA [m^2^] ^d^	CLCR_CG_ABW_ [mL/min]
**(1)** Nonobese, healthy renal function	22.6	70.0 ^I^	52.6	17.4	24.8	1.85	90 ^III^
**(2)** Morbidly obese, healthy renal function	40.0 ^II^	127	76.8	50.2	39.5	2.49	130 ^IV^
**(3)** Nonobese, renally impaired	22.6	70.0	52.6	17.4	24.8	1.85	45.0 ^V^
**(4)** Nonobese, hyperfiltration	22.6	70.0	52.6	17.4	24.8	1.85	130 ^VI^

Calculated via ^a^ [40], ^b^ difference between total body weight and lean body weight, ^c^ fat mass/total body weight 100%, ^d^ calculated by [41]. ^I^ Standard body weight, ^II^ lower BMI threshold for morbid obesity, ^III^ healthy renal function [42], ^IV^ adjusted healthy renal function by “de-indexation” of ^V^ by BSA, ^V^ lower threshold of CKD stage 3A [43], ^VI^ lower threshold of glomerular hyperfiltration. Abbreviations: BMI: Body mass index, BSA: Body surface area, CLCR_CG_ABW_: Creatinine clearance calculated via Cockcroft-Gault using adjusted body weight.

**Table 2:** Summary of the model-evaluated intravenous dosing regimens of piperacillin/tazobactam.

Dosing Regimen	Dose Piperacillin [g]/ Tazobactam [g]	Dosing Interval [h]	Infusion Length [h]	Loading Dose ^1^ Piperacillin [g]/ Tazobactam [g]	Daily Dose Piperacillin [g]/ Tazobactam [g]
**“Standard dosage 1” ^2^**	4/0.5	6	0.5	-	16/2
**“Standard dosage 2” ^2^**	4/0.5	8	4	-	12/1.5
**“High dosage” ^2^**	4/0.5	6	3	-	16/2
**“Continuous infusion”**	24/3	24	24	4/0.5	28/3.5 ^3^

^1^ Infusion over 0.5 h; ^2^ EUCAST.org, accessed 1 June 2021; ^3^ For continuous infusion treatment at day 1, the initial loading dose is included.

**Table 3:** Patient-specific characteristics of obese and nonobese patients.

Characteristic	Full Population (*n* = 30) *	Obese Subpopulation (*n* = 15) *	Nonobese Subpopulation (*n* = 15) *
Sex, male	12 (40.0%)	6 (40.0%)	6 (40.0%)
Total body weight [kg]	96.0 (77.0–123)	122 (109–147)	75.0 (67.0–84.0)
Lean body weight [kg] ^a^	55.5 (46.9–71.8)	64.7 (54.5–83.3)	45.8 (42.4–61.1)
Fat mass [kg] ^b^	39.3 (25.2–63.3)	64.2 (53.0–72.3)	25.2 (22.8–27.8)
Percent fat mass, % ^c^	39.9 (34.4–48.6)	48.6 (46.2–53.7)	34.0 (27.4–38.5)
BMI [kg/m^2^]	33.4 (26.5–44.8)	45.7 (40.1–48.3)	26.4 (24.7–28.1)
Serum creatinine conc. [µmol/L]	70.6 (59.2–86.0)	79.1 (61.0–88.0)	70.0 (57.6–86.0)
CLCR_CG_ABW_ [mL/min]	110 (82.1–130)	131 (113–144)	89.8 (75.1–106)
Serum albumin conc. [g/L]	45.6 (43.1–46.9)	45.6 (44.0–46.9)	45.9 (42.1–47.7)
Total bilirubin conc. [µM]	6.20 (3.70–9.00)	7.70 (4.30–11.6)	5.90 (3.50–6.30)
Arterial hypertension	17 (56.7%)	14 (93.3%)	3 (20.0%)
Diabetes mellitus type 2	6 (20.0%)	4 (26.7%)	2 (13.3%)
Steatohepatitis	8 (26.7%)	8 (53.3%)	0 (0.00%)
Vasopressors ^d^	18 (60.0%)	8 (53.3%)	10 (66.7%)

* Entries are median (25th to 75th percentile) or count (%) ^a^ Calculated via [40] ^b^ Calculated as the difference between total body weight and lean body weight ^c^ Calculated as fat mass/total body weight 100%. ^d^ Noradrenaline or cafedrine/theodranenaline Abbreviations: BMI: Body mass index; CLCR_CG_ABW_: Creatinine clearance calculated via Cockcroft-Gault using adjusted body weight; conc.: Concentration.

## Data Availability

The datasets generated during and/or analysed during the current study are available from the corresponding author on reasonable request.

## Supplementary Materials

The following are available online at https://www.mdpi.com/article/10.3390/pharmaceutics13091380/s1. Figure S1: Relative frequency of minimum inhibitory concentrations for piperacillin/tazobactam for five different pathogens commonly treated with piperacillin/tazobactam. Figure S2: Observed individual piperacillin fraction unbound in plasma for obese and nonobese patients. Figure S3: Observed body mass index versus lean body weight for obese and nonobese patients. Figure S4: Observed individual piperacillin concentration-time profiles in plasma and interstitial space fluid of subcutaneous adipose tissue for both catheters for obese and nonobese patients on linear and semi-logarithmic scale. Table S1: Key models in the population pharmacokinetic model development., Table S2: Parameter estimates (pharmacokinetic parameters for unbound piperacillin and microdialysis methodology-related parameters) including sampling importance resampling results of the final model of piperacillin in obese and nonobese patients. Figure S5: Distribution of observed individual piperacillin maximum and minimum concentrations in plasma and interstitial space fluid of subcutaneous adipose tissue for both catheters for obese and nonobese patients. Figure S6: Random-effects parameter values (η) for structural pharmacokinetic parameters of the final pharmacokinetic model of piperacillin versus the identified continuous covariates. Figure S7: Basic goodness-of-fit plots with observed and predicted piperacillin concentrations on a log-scale for the final pharmacokinetic model for the three matrices: Microdialysate, retrodialysate and plasma (total and unbound). Figure S8: Visual predictive check (*n* = 1000 simulations) for the final pharmacokinetic model for total plasma piperacillin concentrations, unbound plasma concentrations, microdialysate concentrations, and retrodialysate concentrations (log-log scale) for obese and non-obese patients. Figure S9: Pharmacokinetic parameter estimates after imputation of retroperfusate concentrations in one obese patient (both microdialysis catheters) via four different imputation strategies. Figure S10: Working steps towards quantification of differences in plasma and target-site exposure and probability of target attainment of piperacillin/tazobactam. Figure S11: Simulated unbound piperacillin concentration-time profiles in plasma and in the interstitial space fluid of the subcutaneous adipose tissue (target-site) and target-site: plasma %*f*T_>MIC_ ratio for four different reference patients (1)–(4), defined in Table 1 in the main manuscript. Figure S12: Ratio of unbound piperacillin concentration in plasma:target-site over time after an intravenous 24 g/3 g piperacillin/tazobactam continuous infusion over 24 h following a 4 g/0.5 g piperacillin/tazobactam 0.5 h i.v. loading dose. Figure S13: Probability of target attainment based on the PK/PD target %*f*T_>MIC_ = 50 versus lean body weight and fat mass in plasma (left panel) and target-site (right panel) stratified by (i) glomerular filtration rate according to CKD stages (moderate: 45.0–59.0 mL/min, mild: 60.0–98 mL/min, normal: >90 mL/min) or glomerular hyperfiltration (≥130 mL/min) covered by this study and (ii) MIC (2.00, 4.00, 8.00, 16.0 mg/L) after four different i.v. infusion regimens. Figure S14: Probability of target attainment based on the PK/PD target %*f*T_>MIC_ = 98 versus lean body weight and fat mass in plasma (left panel) and target-site (right panel) stratified by (i) glomerular filtration rate according to CKD stages (moderate: 45.0–59.0 mL/min, mild: 60.0–98 mL/min, normal: >90 mL/min) or glomerular hyperfiltration (≥130 mL/min) covered by this study and (ii) MIC (2.00, 4.00, 8.00, 16.0 mg/L) after four different i.v. infusion regimens.
