# Supplementary material for: Similar Piperacillin/Tazobactam Target Attainment in Obese versus Nonobese Patients despite Differences in Interstitial Tissue Fluid Pharmacokinetics
Source: Pharmaceutics. 2021 Aug 31;13(9):1380. doi: 10.3390/pharmaceutics13091380 (PMC8464843; doi:10.3390/pharmaceutics13091380)
Supplement: Supplementary file 1 [file pharmaceutics-13-01380-s001.zip › pharmaceutics-1315035-SI.pdf]

pharmaceutics

MDPI

# Supplementary Materials: Similar Piperacillin/Tazobactam Target Attainment in Obese versus Nonobese Patients despite Differences in Interstitial Tissue Fluid Pharmacokinetics

**David Busse, Philipp Simon, David Petroff, Christoph Dorn, Lisa Schmitt, Davide Bindellini, Alexander Kratzer, Arne Dietrich, Markus Zeitlinger, Wilhelm Huisinga, Robin Michelet, Hermann Wrigge and Charlotte Kloft**

## Model Development

Interindividual variability was included on the PK parameters assuming a log-normal distribution:

$$\theta_i = \theta_{pop} \cdot e^{\eta_i}$$

$\theta_i$ is the individual PK parameter for individual i, $\theta_{pop}$ the population parameter of the PK parameter, and $\eta_i$ is the between-patient random-effects parameter on individual i. $\eta_i$ was assumed to be normally distributed with a mean of 0 and a variance of $\sigma^2$. Additive, proportional and additive/proportional combined models of residual unexplained variability were evaluated.

Statistical comparison between nested models with additional covariates was done using the likelihood ratio test (Δobjective function value ≥ 3.84 at $\alpha = 0.05$ and 1 degree of freedom) [1] and the Akaike information criterion [2] for nested and non-nested models, respectively. Model selection was based on plausibility and precision of parameter estimates, quantified by sampling importance resampling (SIR) ($n_{samples}$ = 1000, 1000, 1000, 2000, 2000, $n_{resamples}$=200, 400, 500, 1000, 1000) [3].

Nonlinear mixed-effects model development and simulations were performed in NONMEM v7.4.3 (Icon Development Solutions, Ellicott City, MD, USA) accessed with PsN v4.8.1 through Pirana v2.9.6 (Certara, Princeton, NJ, USA). RStudio v1.2.1335 (RStudio, Boston, MA, USA) was used for dataset preparation and post-processing of results.

The physiologically-motivated impact of LBW [4] *and* fat mass (FM = total body weight − LBW) on $V_2$ was implemented as follows:

$$V_{2,i} = V_{2,pop} \cdot \left( R \cdot \frac{LBW_i}{LBW_{pop}} + (1 - R) \cdot \frac{FM_i}{FM_{pop}} \right)^1 \cdot e^{\eta_i}$$

$V_{2,i}$ is the individual peripheral volume for individual i, $V_{2,pop}$ is the population mean of this volume, and $\eta_i$ is the between-patient random-effects parameter on individual i. $FM_i$ and $LBW_i$ are individual and $FM_{pop}$ and $LBW_{pop}$ patient medians of the respective body size descriptors. R is a weighting parameter describing the proportion of $V_{2,pop}$ scaled by LBW. The estimate of R (68.4% [48.1%, 90.4%]) indicated that the impact of LBW on $V_{2,pop}$ was much higher than that of FM (1−R = 31.6%).

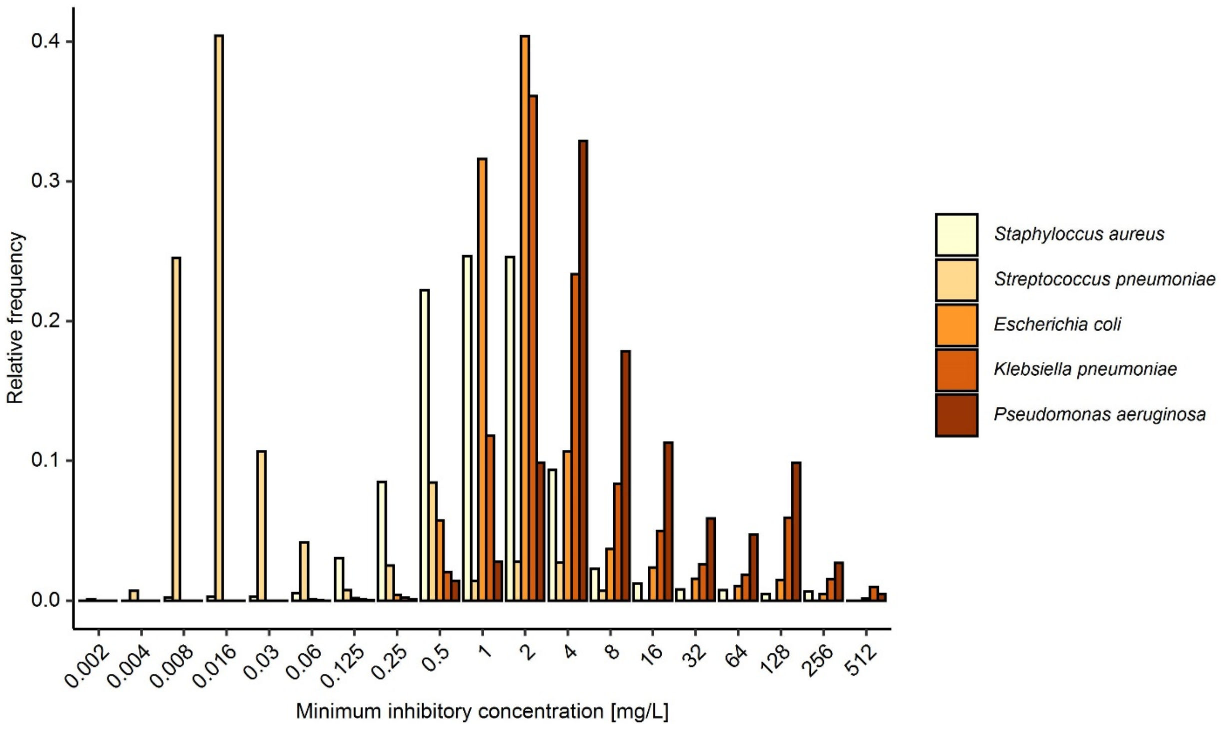


**Figure S1.** Relative frequency of minimum inhibitory concentrations for piperacillin/tazobactam (for determination of the piperacillin minimum inhibitory concentration the tazobactam concentration has been fixed at 4 mg/L: EUCAST.org, accessed 2021-06-01) for five different pathogens commonly treated with piperacillin/tazobactam.

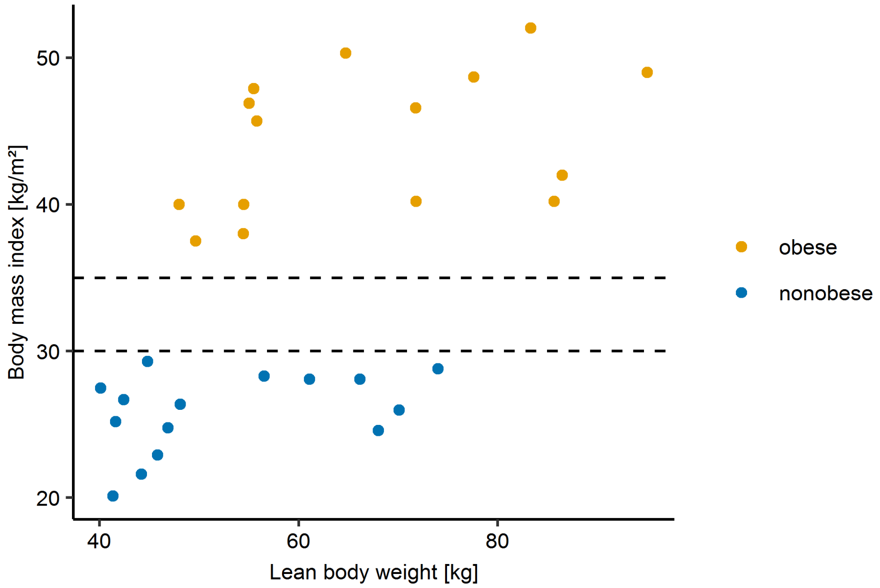


**Figure S2.** Observed individual piperacillin fraction unbound in plasma for obese ($n$ = 15, orange) and nonobese patients ($n$ = 15, blue). Dashed horizontal line: Median fraction unbound in plasma. Abbreviations: $CLCR_{CG\_ABW}$: Creatinine clearance calculated via Cockcroft-Gault based on adjusted body weight.

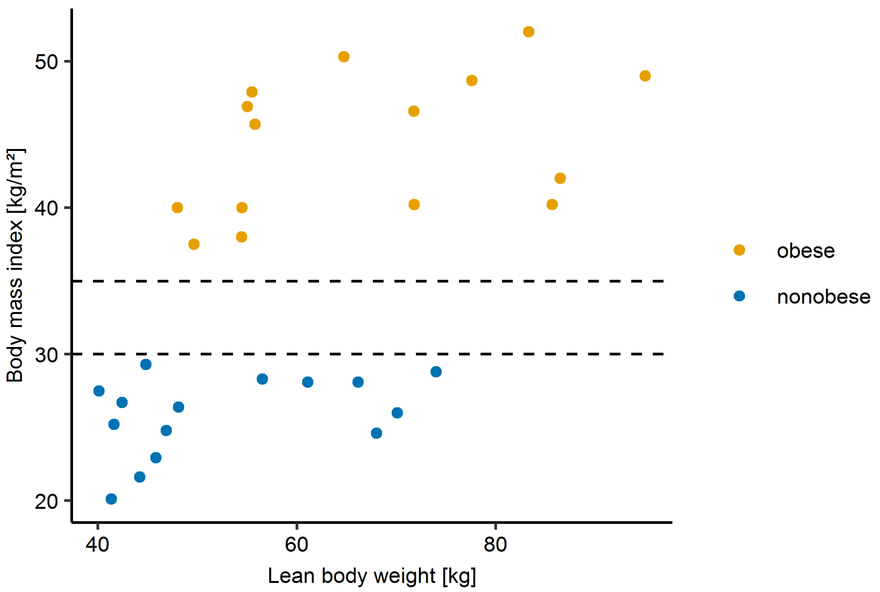


\

**Figure S3.** Observed body mass index versus lean body weight [4] for obese (*n* = 15, orange) and nonobese patients (*n* = 15, blue). Dashed horizontal lines: Range for exclusion criteria for nonobese patient group (body mass index < 30 kg/m²) and obese patient group (body mass index ≥ 35 kg/m²).

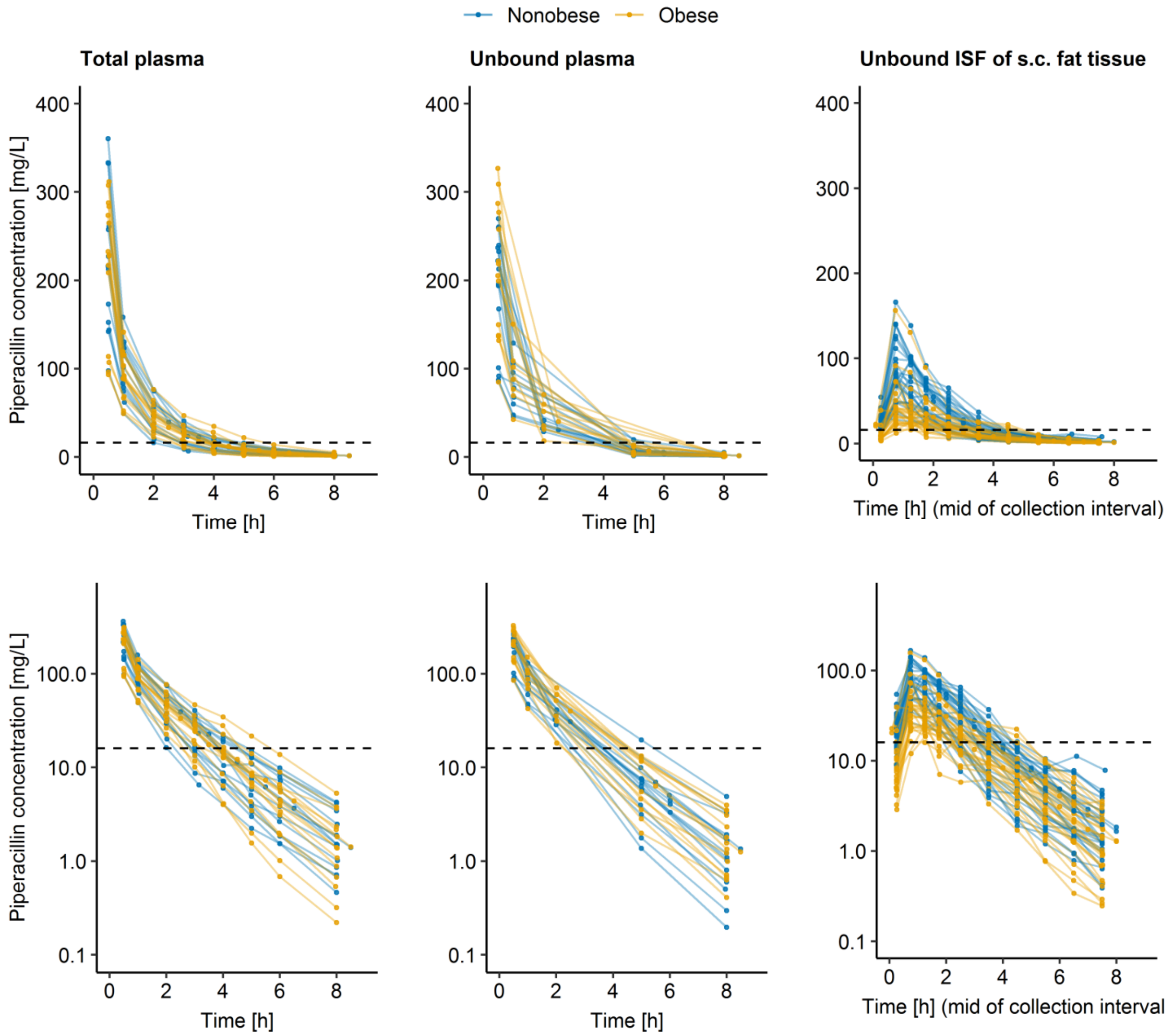

**Figure S4.** Observed **i**ndividual piperacillin concentration-time profiles in plasma (total concentration: left, unbound concentration: middle) and interstitial space fluid of subcutaneous adipose tissue for both catheters (unbound concentration, right) for obese (*n* = 15, orange) and nonobese patients (*n* = 15, blue) on linear (upper panel) and semi-logarithmic scale (lower panel). ISF concentrations are displayed at the mid-time of the respective collection intervals. Dashed line: Minimum inhibitory concentration=16 mg/L.
Abbreviations: ISF: Interstitial space fluid; s.c.: Subcutaneous.

**Table S1.** Key models in the population pharmacokinetic model development.

| Development Step | Model Description | AIC | Selection |
|---|---|---|---|
| 1. Structural model | 2-CMT model, ISF associated with central CMT | -[1] | |
| | 2-CMT model, ISF associated with peripheral CMT | 3801 | |
| | 3-CMT **mammillary** model, ISF associated with "shallow" peripheral CMT | 3565 | x |
| | 3-CMT **serial** model, ISF associated with "shallow" peripheral CMT | 3802 | |
| 2. Impact of body size descriptors on piperacillin distribution and elimination (investigated via allometric scaling) | Ideal body weight, fixed exponents | 3590 | |
| | est. exponents | 3590 | |
| | Lean body weight, fixed exponents | 3575 | |
| | est. exponents | 3575 | |
| | Total body weight, fixed exponents | 3571 | |
| | est. exponents[2] | 3559 | |
| | Body mass index, fixed exponents | 3575 | |
| | est. exponents | 3575 | |
| | Adjusted body weight, fixed exponents | 3574 | |
| | est. exponents | 3576 | |
| | LBW/FM | 3571 | x |
| | NFM[2] | 3571 | |
| 3. Impact of renal function on piperacillin clearance | Ideal body weight-based $CLCR_{CG}$ | 3569 | |
| | Lean body weight-based $CLCR_{CG}$ | 3568 | |
| | Total body weight-based $CLCR_{CG}$ | 3568 | |
| | Adjusted body weight-based $CLCR_{CG}$ | 3568 | x |
| | ↪additional impact of lean body weight | 3569 | |
| | ↪additional impact on fraction unbound | 3571 | 3571 |
| | de-indexed eGFR by MDRD | 3568 | |
| | de-indexed eGFR by CKD-EPI | 3568 | |
| 4. Impact of anesthesia-related hemodynamic | Presence of anesthesia/surgery on tissue factor | 3548 | x |

| | | |
|---|---|---|
| changes on piperacillin PK parameters | Mean arterial blood pressure on flows | 3549 |

[1] Non-convergence of estimation algorithm

[2] Estimated exponents for flows imprecise (0.354, 95%CI=(−0.02; 0.728))

[3] Estimated fat mass scaling factor imprecise (0.675, 95%CI=(−0.301; 1.651))

Abbreviations: AIC: Akaike information criterion; CKD-EPI: Chronic Kidney Disease Epidemiology Collaboration formula; $CLCR_{CG}$: Creatinine clearance calculated via the Cockcroft-Gault formula; CMT Compartment; eGFR: Estimated glomerular filtration rate, est:. Estimated; ISF: Interstitial space fluid of subcutaneous fat tissue; LBW/FM: Allometric scaling lean body weight (central volume of distribution and flows) and lean body weight and fat mass (peripheral volume of distribution associated with ISF of subcutaneous fat tissue); MDRD: Modification of Diet in Renal Disease formula; NFM: Normal fat mass based allometric scaling; PK: Pharmacokinetics.

**Table S2.** Parameter estimates (pharmacokinetic parameters for unbound piperacillin and microdialysis methodology-related parameters) including sampling importance resampling results of the final model of piperacillin in obese and nonobese patients.

| Parameter [unit] | Final model<br>Estimate (RSE[1], %) | Sampling Importance Resampling[2]<br>Median | <br>95% CI |
|---|---|---|---|
| **Structural and covariate parameters** | | | |
| CL [L/h] for median $CLCR_{CG\_ABW}$ of 110 mL/min | 16.4 (6.00) | 16.4 | (15.2; 17.9) |
| Impact $CLCR_{CG\_ABW}$ on CL[3] [L·h$^{-1}$/10 mL/min] | 0.583 (41.0) | 0.597 | (0.539; 0.567) |
| $V_1$ for median LBW of 55.6 kg[4] [L] | 11.8 (9.00) | 11.9 | (10.4; 13.6) |
| $Q_1$ for median LBW of 55.6 kg[4] [L/h] | 6.47 (16.0) | 6.49 | (4.95; 8.11) |
| $V_2$ for median LBW of 55.6 kg[4] and FM of 39.3 kg[5] [L] | 3.58 (13.0) | 3.59 | 82.73; 4.53) |
| $Q_2$ for median LBW of 55.6 kg[4] [L/h] | 5.80 (17.0) | 5.77 | (4.57; 7.07) |
| $V_3$ for median LBW of 55.6 kg[4] [L] | 4.91 (16.0) | 4.92 | (4.05; 6.07) |
| $F_u$, % | 89.9 (1.50) | 89.9 | (87.8; 91.9) |
| R, % | 68.4 (41.0) | 67.9 | (48.1; 90.4) |
| TF, % | 70.1 (6.00) | 69.9 | (62.3; 75.4) |
| $TF_{anesthesia}$, % | 14.7 (25.0) | 14.6 | (10.3; 19.0) |
| $RR_{OBE}$, % | 29.1 (9.00) | 29.1 | (24.4; 34.4) |
| $RR_{NOBE}$, % | 59.9 (7.00) | 59.7 | (53.9; 65.7) |
| **Interindividual variability parameters, %CV** | | | |
| CL | 32.2 (14.0) | 31.6 | (25.8; 39.1) |
| $V_1$ | 49.4 (13.0) | 49.6 | (41.1; 59.3) |
| $V_2$ | 53.9 (15.0) | 54.7 | (41.5; 69.9) |
| $V_3$ | 23.6 (16.0) | 23.8 | (17.8; 29.1) |
| **Microdialysis technique-related variability parameters** | | | |
| $\sigma^2_{Interindividual}$ RR | 0.417 (37.0) | 0.417 | (0.294; 0.548) |
| $\sigma^2_{Intercatheter}$ RR | 0.400 (30.0) | 0.402 | (0.312; 0.502) |
| $\sigma^2_{Intracatheter}$ RR | 0.837 (28.0) | 0.838 | (0.745; 0.942) |
| **Residual variability parameters** | | | |
| $\sigma_{proportional,\ plasma}$, %CV | 10.2 (11.0) | 10.2 | (9.35; 11.4) |
| $\sigma_{additive,\ plasma}$ [mg/L] | 0.115 (26.0) | 0.115 | (0.0823; 0.114) |
| $\sigma_{proportional,\ micrdialysis}$, %CV | 22.6 (5.00) | 22.7 | (21.2; 24.5) |
| $\sigma_{proportional,\ retrodialysis}$, %CV | | 2.90[6] | |

[1] RSE of random-effects are reported on approximate standard deviation scale

[2] $n_{samles}$=1000, 1000, 1000, 2000, 2000; $n_{resamples}$=200,400,500,1000,1000

[3] Centred to median $CLCR_{CG\_ABW}$ in overall population (110 mL/min)

[4] Allometrically scaled with LBW centered to median in overall population (55.6 kg) with exponent of 1 for volumes and 0.75 for flows

[5] Allometrically scaled with FM centered to median in overall population (39.3 kg) with exponent of 1 [6]Fixed to interassay variability

Abbreviations: CI: Confidence interval; CL: Clearance; $CLCR_{CG\_ABW}$: Creatinine clearance calculated via Cockcroft-Gault based on ABW; CV: Coefficient of variation; FM: Fat mass; $F_u$: Fraction unbound; LBW: Lean body weight; NOBE: Nonobese population; OBE: Obese population; $Q_{1,2}$: Intercompartmental flows; R: Weighting parameter describing the

proportion of $V_2$ scaled by LBW; RSE: Relative standard error; $RR_{OBE}/RR_{NOBE}$: Relative recovery for obese/nonobese patients; $V_1$: Central volume of distribution; $V_2$: "Shallow" peripheral volume of distribution associated with the target-site; $V_3$: "Deep" peripheral volume of distribution; $\sigma$: Residual unexplained variability; $\sigma^2$: Variance associated with retrodialysis.

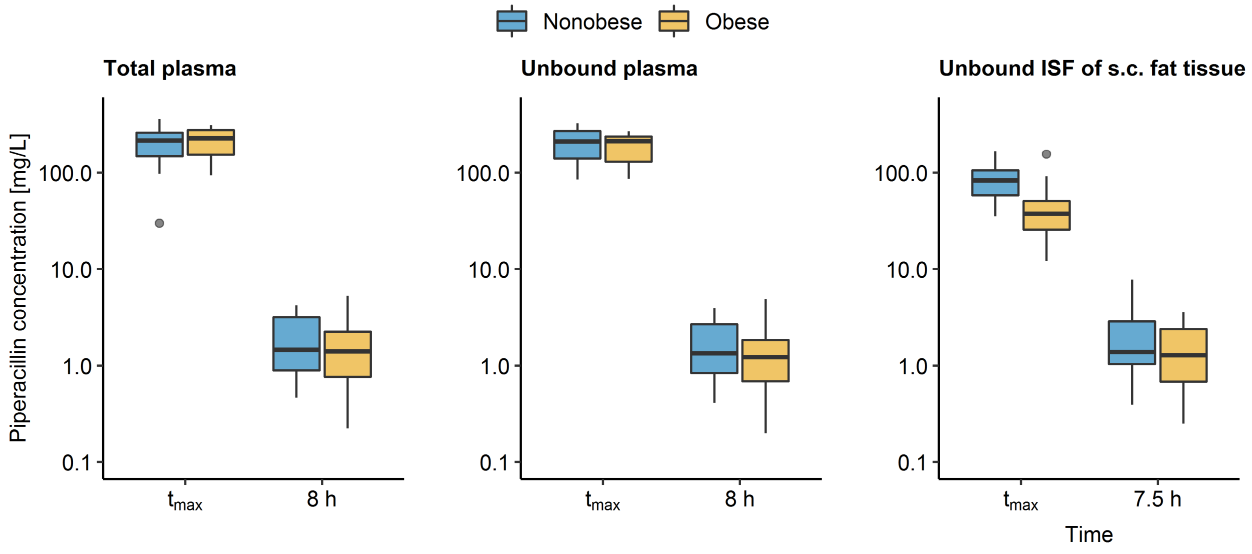


**Figure S5.** Distribution of observed individual piperacillin maximum and minimum concentrations in plasma (total concentration: left; unbound concentration: mid) and interstitial space fluid of subcutaneous adipose tissue for both catheters (unbound concentration, right) for obese ($n$ = 15, orange) and nonobese patients ($n$ = 15, blue). ISF concentrations are displayed at the mid-time of the respective collection intervals.
Abbreviations: ISF: Interstitial space fluid; s.c.: Subcutaneous; $t_{max}$: Time of maximum piperacillin concentration.

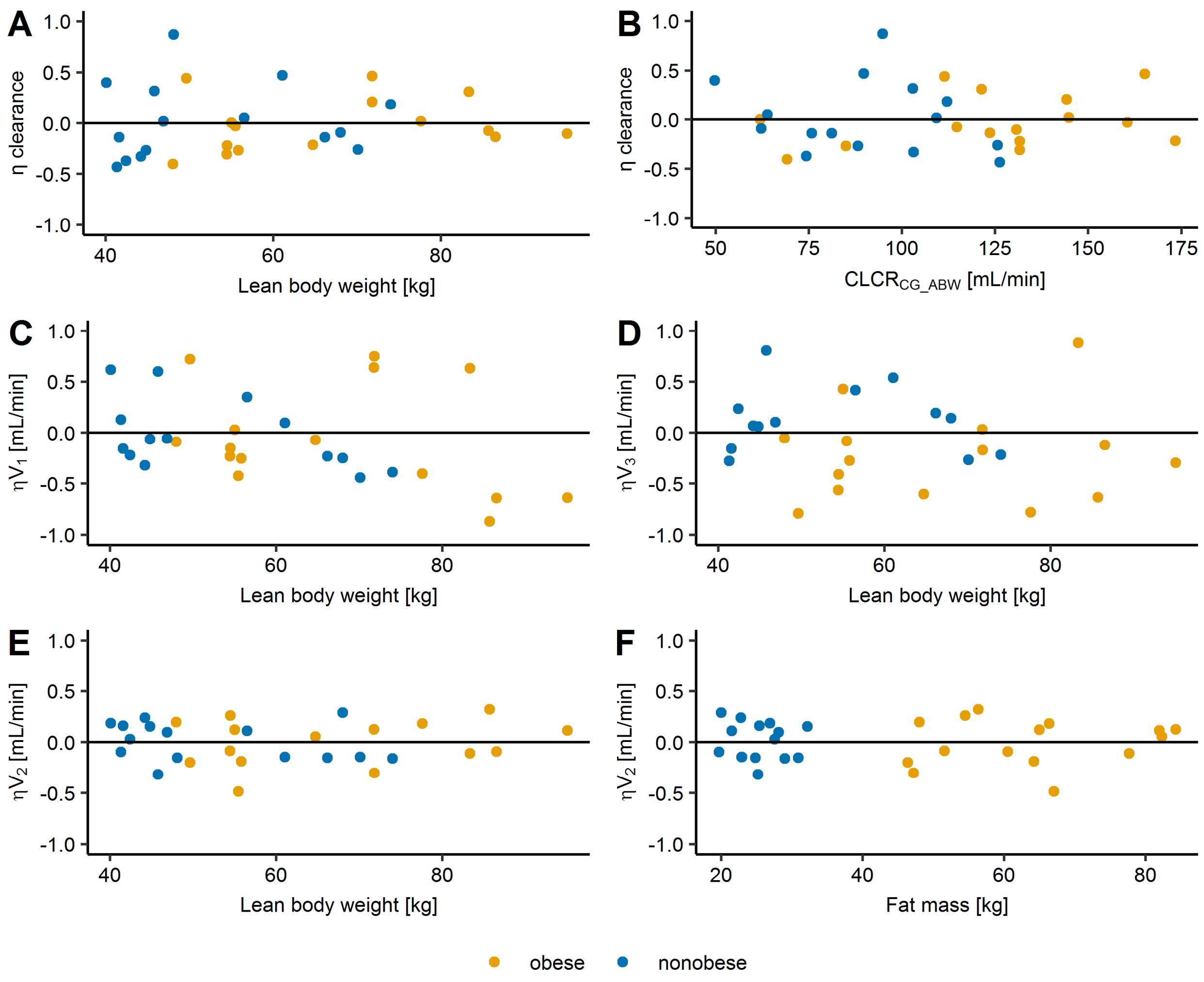


**Figure S6.** Random-effects parameter values (η) for structural pharmacokinetic parameters of the final pharmacokinetic model of piperacillin versus the identified continuous covariates. See Table S3 for abbreviations.

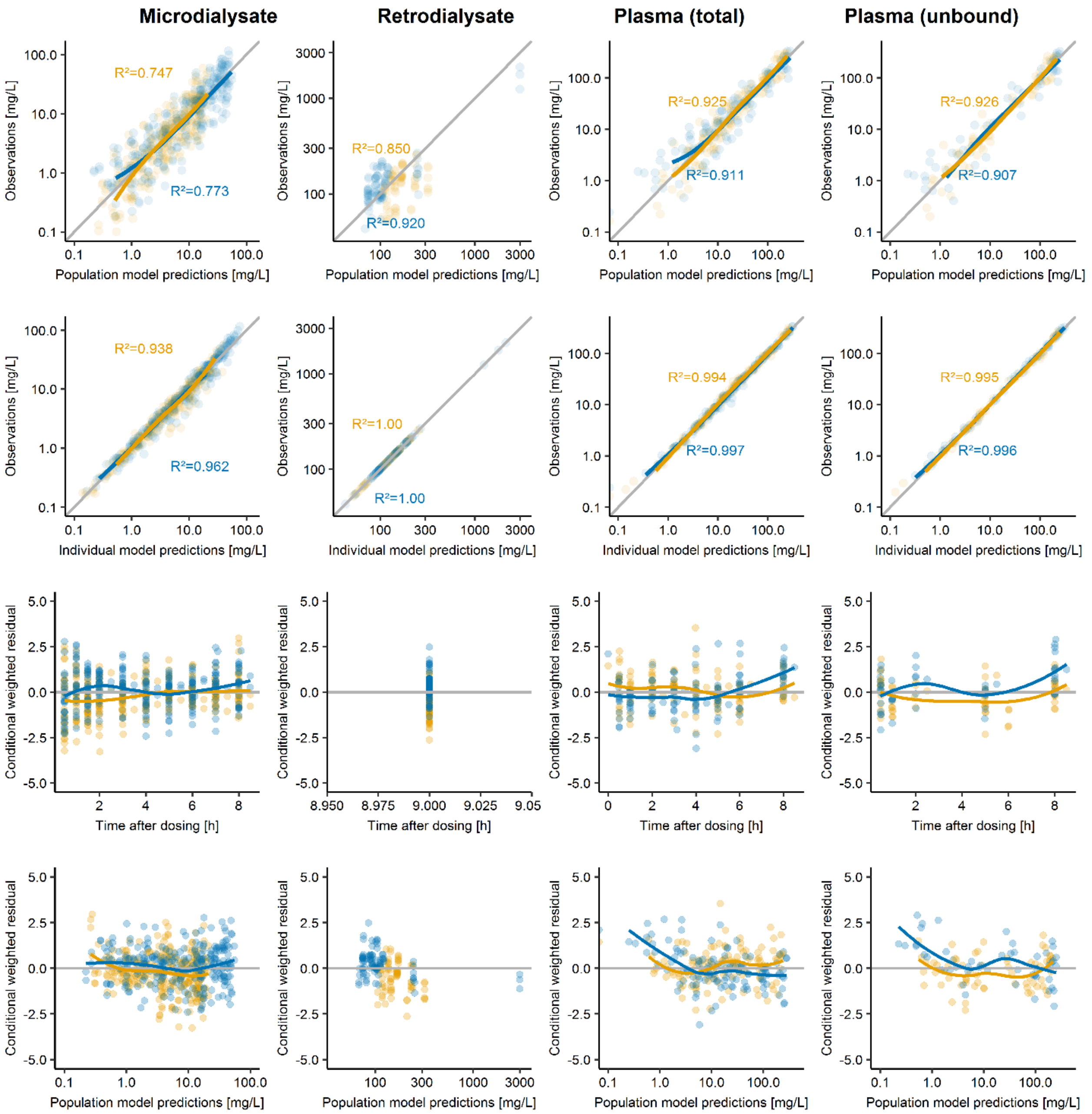


**Figure S7.** Basic goodness-of-fit plots with observed and predicted piperacillin concentrations on a log-scale for the final pharmacokinetic model for the three matrices: Microdialysate, retrodialysate and plasma (total and unbound). Orange dots: obese patients; blue dots: nonobese patients; gray line: Line of identity; orange line: Loess smoother for obese patients; blue line: Loess smoother for nonobese patients.

Abbreviations: $R^2$: Coefficient of determination.

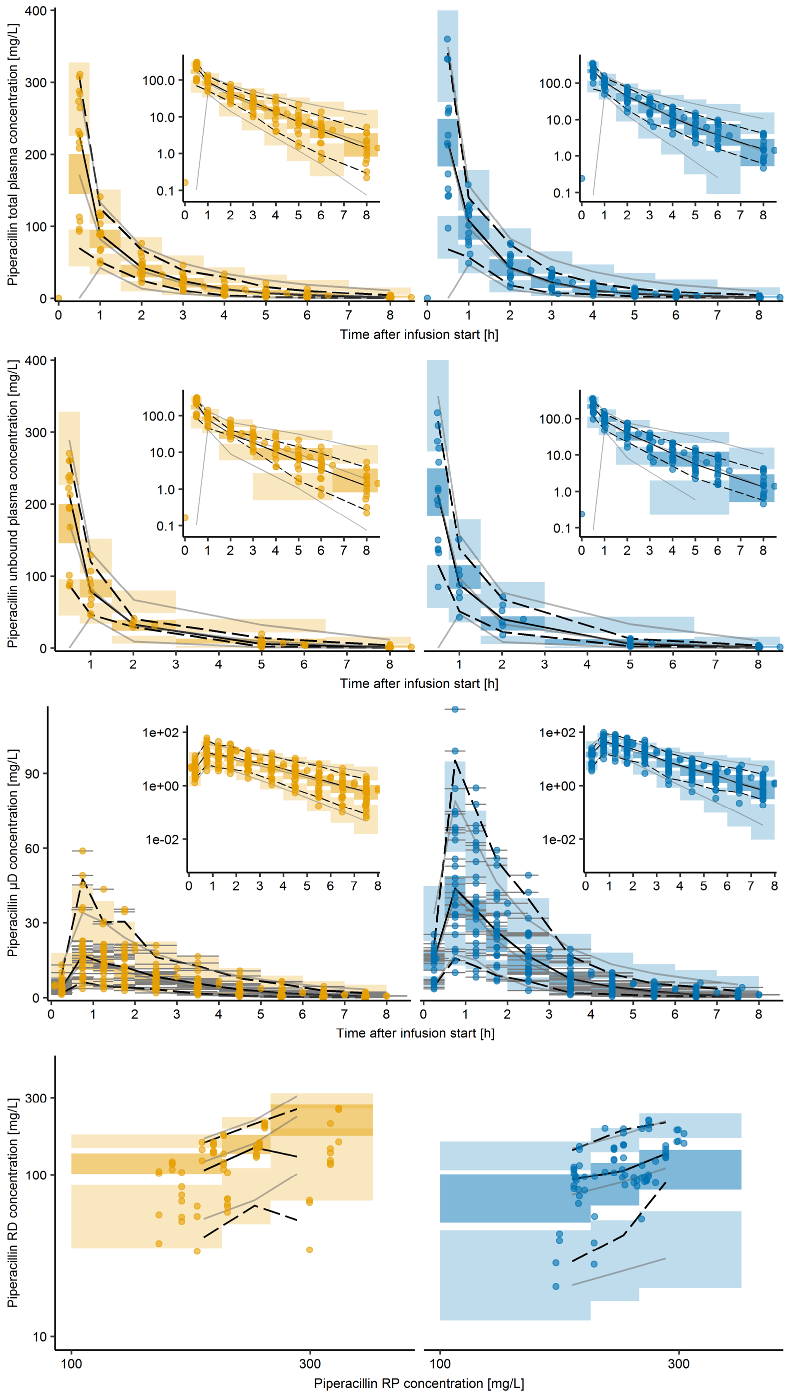


**Figure S8.** Visual predictive check (*n* = 1000 simulations) for the final pharmacokinetic model for total plasma piperacillin concentrations (1st panel), unbound plasma concentrations (2nd panel), microdialysate concentrations (3rd panel), and retrodialysate concentrations (4th panel, log-log scale) for obese (orange) and non-obese patients (blue). Circles: Observed piperacillin concentrations; Black lines: 5th, 95th percentile (dashed), 50th percentile (solid) of the observed data; Grey solid lines: 5th, 50th and 95th percentile of simulations. Shaded areas: 95% confidence interval around 5th, 50th and 95th percentile of simulated data. Horizontal grey lines: Microdialysate collection interval. Plot insets are on semi-logarithmic scale (1st to 3rd panel).

Abbreviations: µD: Microdialysate; RD: Retrodialysate; RP: Retroperfusate.

**Imputation of Missing Retroperfusate Concentration**

The retroperfusate concentration for both microdialysis catheters was missing in one obese patient. The impact of the following imputation strategies on PK parameter estimates was evaluated:

1. Imputation by nominal retroperfusate concentration (200 mg/L)
2. Imputation by median retroperfusate concentration in all patients (234 mg/L)
3. Imputation by 5th percentile of retroperfusate concentrations in all patients (150 mg/L)
4. Imputation by 95th percentile of retroperfusate concentrations in all patients (632 mg/L)

The impact of selecting either of the 4 imputation strategies on PK parameter estimates was low (95% CI overlapped for all PK parameter estimates, Figure S1). Finally, the nominal retroperfusate concentration (200 mg/L; imputation strategy 1) was selected for imputation.

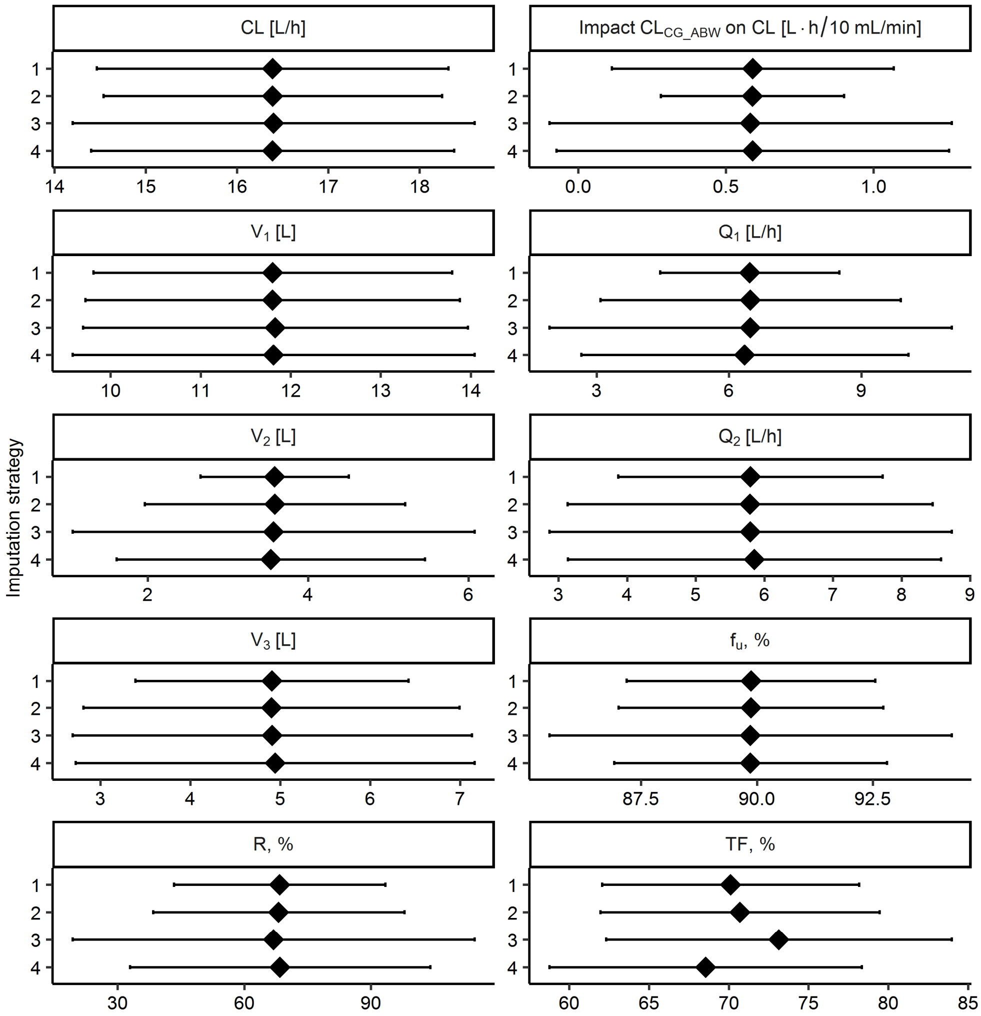


**Figure S9.** Pharmacokinetic parameter estimates after imputation of retroperfusate concentrations in one obese patient (both microdialysis catheters) via four different imputation strategies (1: nominal retroperfusate concentration, 2/3/4: Median/5th percentile/95th percentile of retroperfusate concentration in all patients).
Diamonds: Population parameter estimates; Black horizontal lines: 95%CI of population parameter estimates; Bold number: Selected imputation strategy.

Abbreviations: see Table S3.

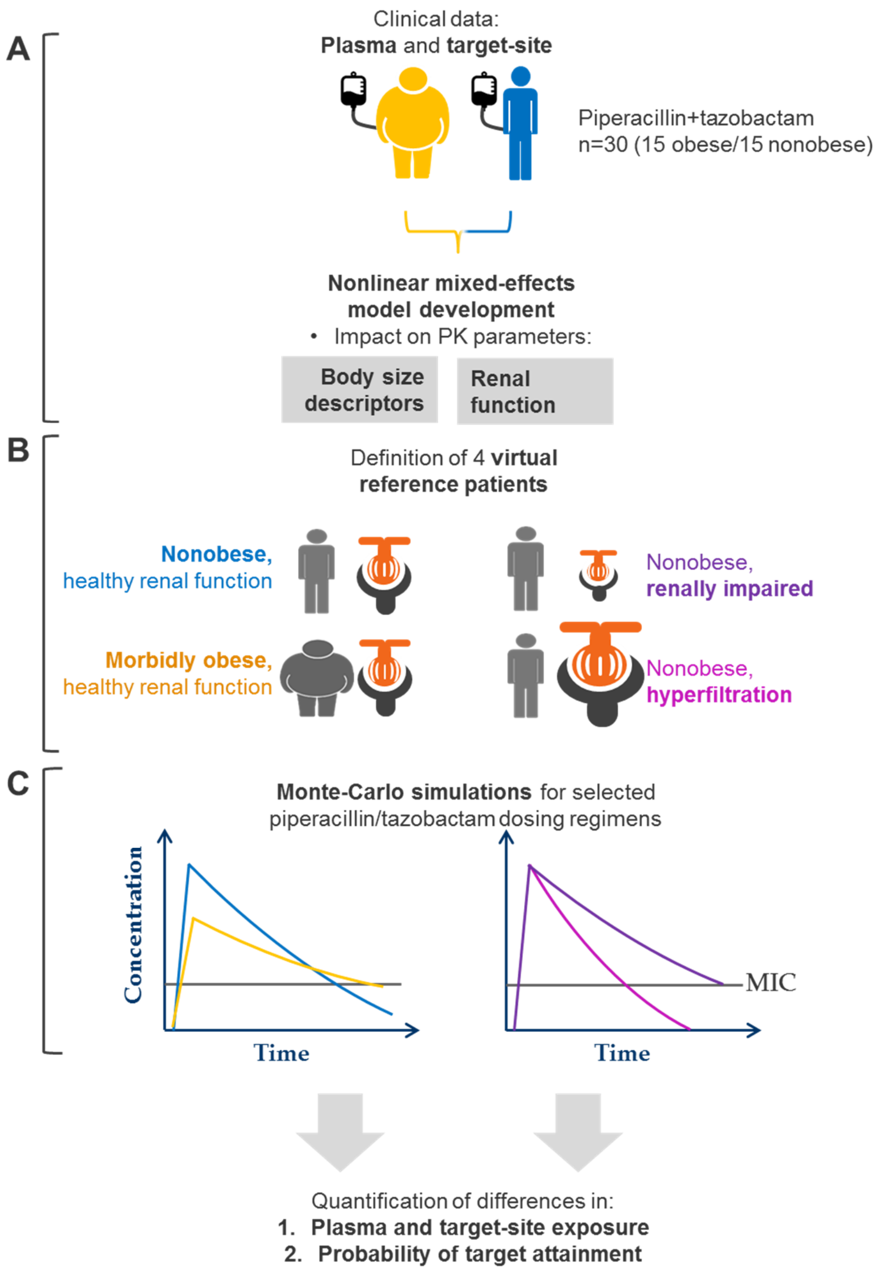


**Figure S10.** Working steps (A: NLME model development, B: Definition of 4 virtual reference patients, C: Monte-Carlo simulations for selected piperacillin/tazobactam dosing regimens) towards quantification of differences in (1.) plasma and target-site exposure and (2.) probability of target attainment of piperacillin/tazobactam (bottom).

Abbreviations: MIC: Minimum inhibitory concentration, PK: Pharmacokinetic(s).

Target-Site Penetration in Obese and Nonobese Patients

The impact of obesity status and renal function on unbound piperacillin exposure was evaluated based on the "standard dosage 1" of piperacillin (Figure S11). Median maximum unbound piperacillin concentrations were lower in plasma and at target-site (−26.5%–−30.0%) in the morbidly obese (BMI = 40.0 kg/m$^2$), healthy renal function reference patient (2) compared to the nonobese (BMI = 22.6 kg/m$^2$), healthy renal function reference patient (1) whereas the minimum piperacillin concentration was higher in (3) versus (1) in both matrices (+37.0%+38.2%).

Higher maximum (+3.76%–+8.66%, Figure 2 lower panel) and minimum unbound piperacillin concentrations (+78.4%–+70.0%) were reached in the nonobese, renally impaired (CKD stage 3A, $CLCR_{CG\_ABW}$=45.0 mL/min) reference patient (3) compared to the nonobese, healthy renal function ($CLCR_{CG\_ABW}$=90.0 mL/min) reference patient (1). Conversely, lower maximum (−5.18%–−7.62%) and minimum unbound piperacillin concentrations (−35.0%–−32.9%, Fig. S11E,F) were reached in the nonobese, hyperfiltration ($CLCR_{CG\_ABW}$=130 mL/min) reference patient (4) compared to reference patient (1).

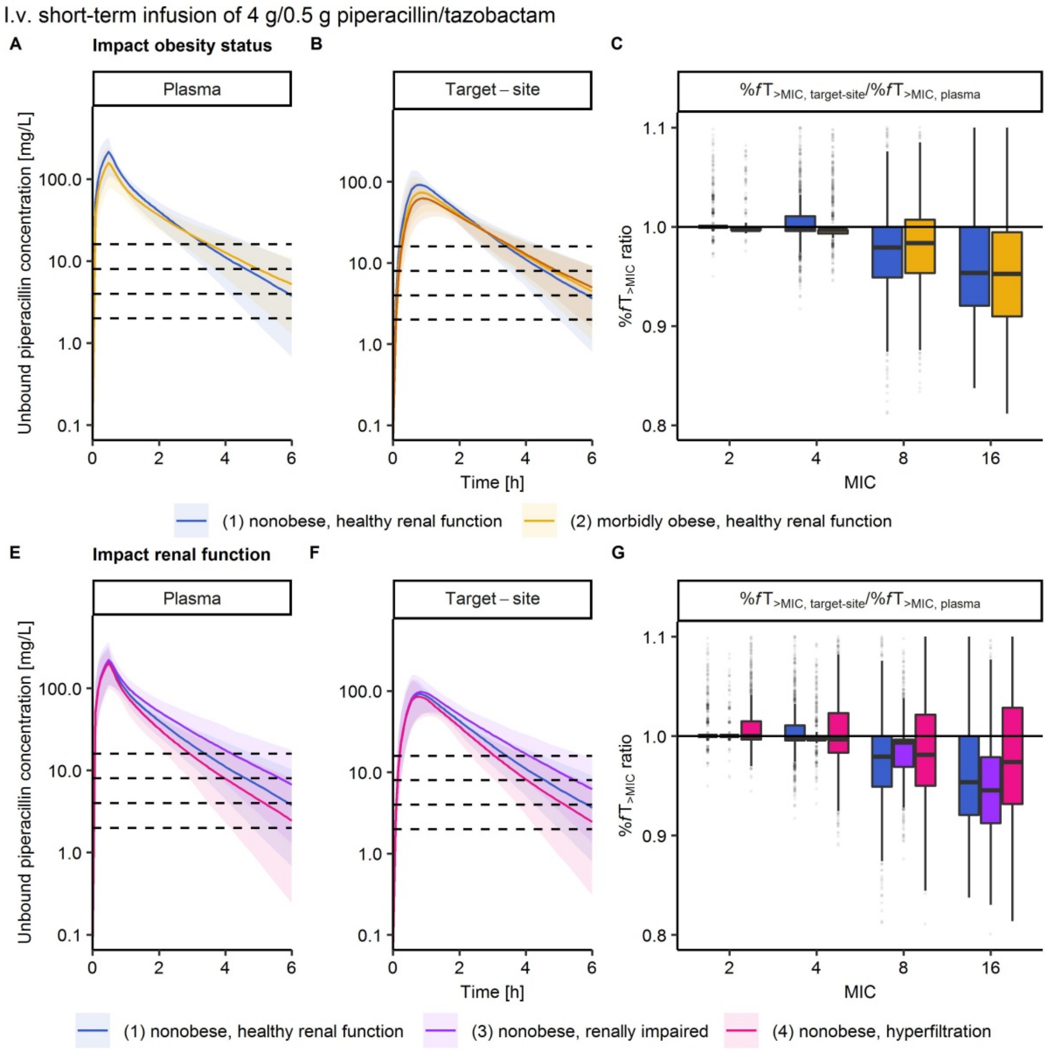


**Figure S11.** Simulated unbound piperacillin concentration-time profiles in plasma (A, E) and in the interstitial space fluid of the subcutaneous adipose tissue (target-site, B, F) and target-site:plasma %$f$T$_{>MIC}$ ratio for four different reference patients (1)–(4), defined in Table 1 in the main manuscript.

Dashed horizontal line: Minimum inhibitory concentration=2–16 mg/L.

Abbreviations: %$f$T$_{>MIC}$: Fraction of time that unbound piperacillin concentrations exceed the minimum inhibitory concentration during 24 h; CLCR$_{CG_ABW}$: Creatinine clearance calculated via Cockcroft-Gault equation based on adjusted body weight.

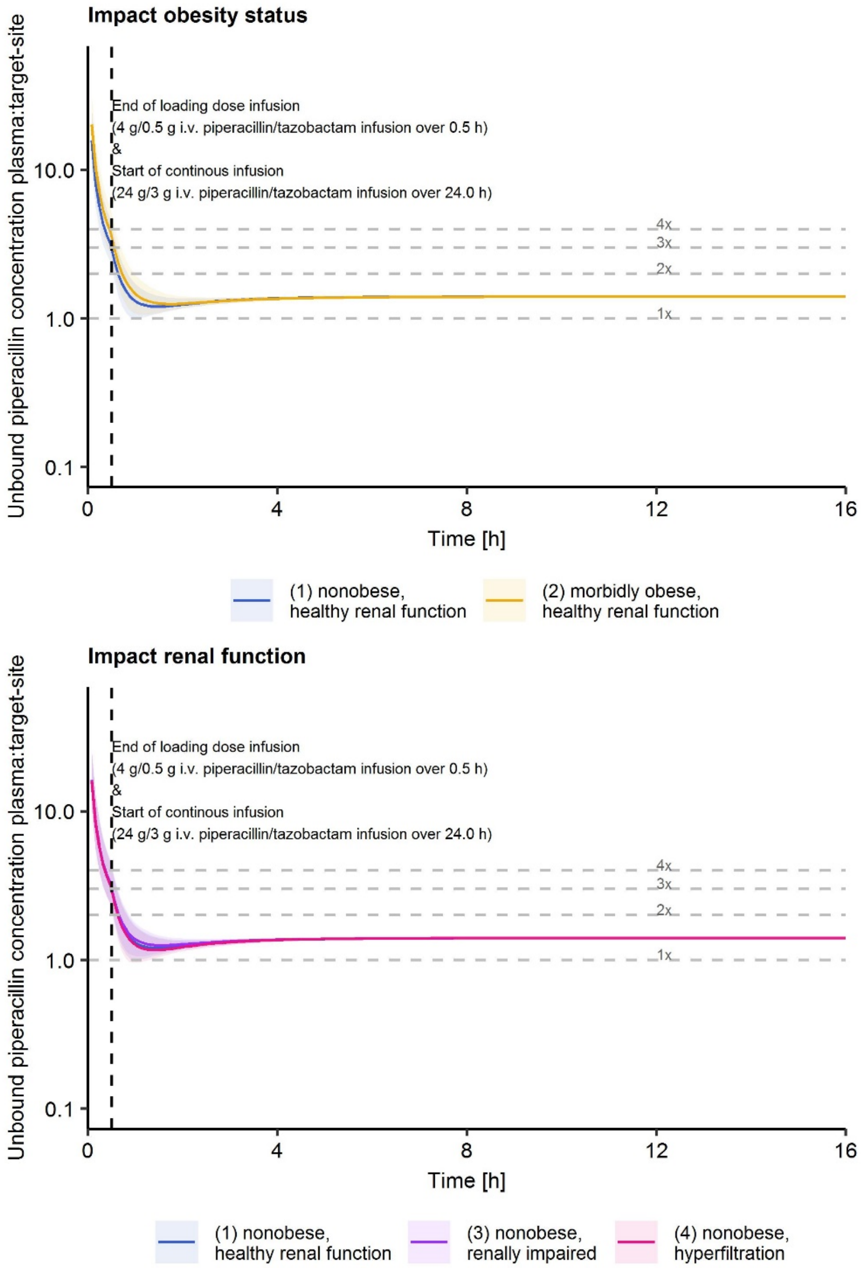


**Figure S12.** Ratio of unbound piperacillin concentration in plasma:target-site over time after an intravenous 24 g/3 g piperacillin/tazobactam continuous infusion over 24 h following a 4 g/0.5 g piperacillin/tazobactam 0.5 h i.v. loading dose. For a description of the five reference patients (1)–(4) see Table 1 in main manuscript.
Dashed grey lines: Multiples of unbound plasma:target-site concentrations.
Abbreviations: i.v.: Intravenous; Loading dose: LD.

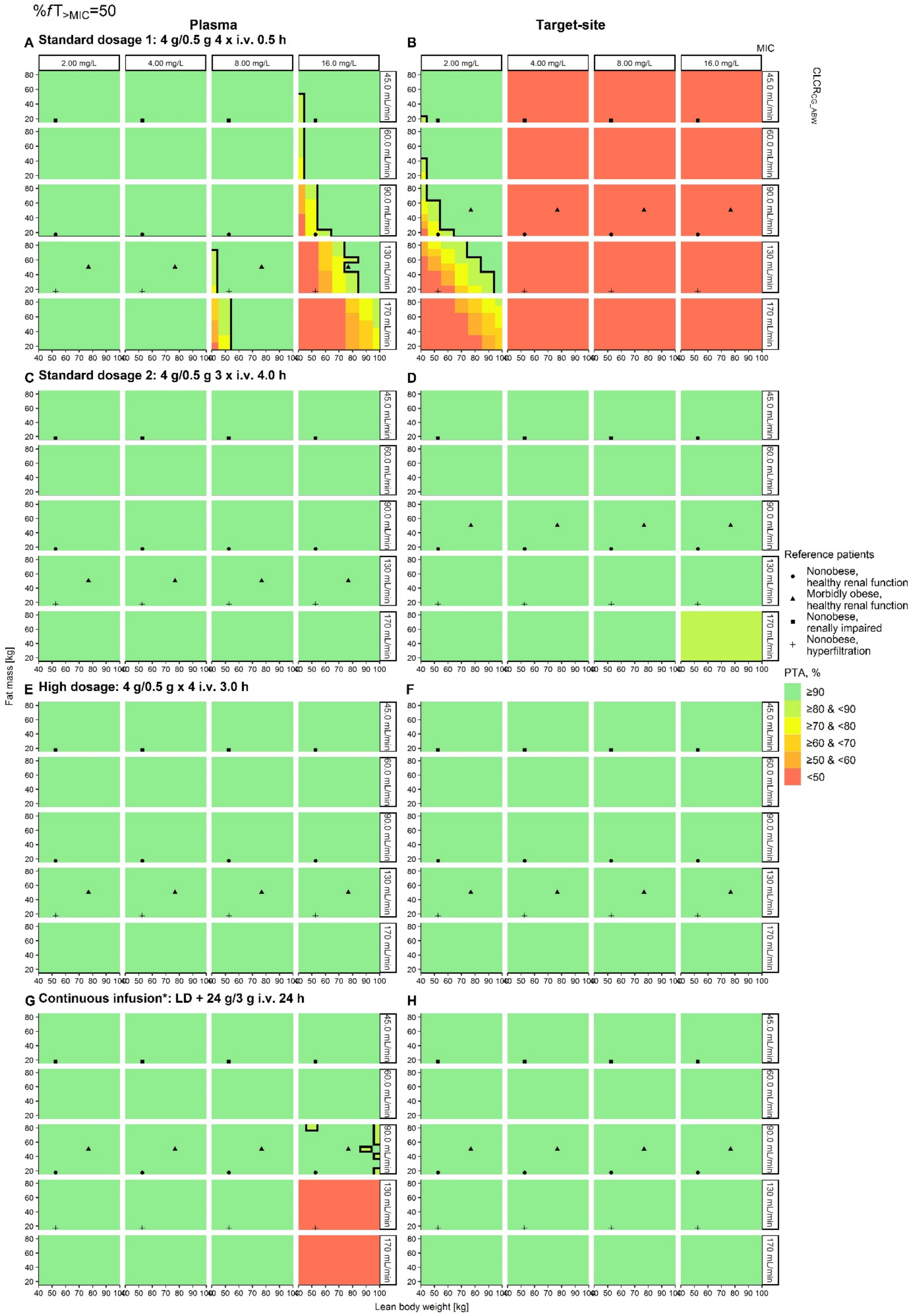


**Figure S13.** Probability of target attainment (PTA) based on the PK/PD target $\%fT_{>MIC}$=50 versus lean body weight and fat mass in plasma (left panel) and target-site (right panel) stratified by (i) glomerular filtration rate according to CKD stages

(moderate: 45.0–59.0 mL/min, mild: 60.0–98 mL/min, normal: >90 mL/min) or glomerular hyperfiltration (≥130 mL/min) covered by this study and (ii) MIC (2.00, 4.00, 8.00, 16.0 mg/L) after four different i.v. infusion regimens.
Asterisk: Pharmacokinetic/pharmacodynamic targets related to 4×MIC in plasma and 1×MIC at target-site; Reference patients: See description Table 1 in main manuscript. Bold black line: separates PTA≥90% (adequate therapy) from PTA<90%.

Abbreviations: %$fT_{>MIC}$: Fraction of time that unbound concentration exceeds MIC over 24 h; CKD: Chronic kidney disease; $CLCR_{CG\_ABW}$: Creatinine clearance calculated via Cockcroft-Gault equation based on adjusted body weight; i.v.: Intravenous; LD: 4 g i.v. loading dose over 0.5 h; MIC: Minimum inhibitory concentration.

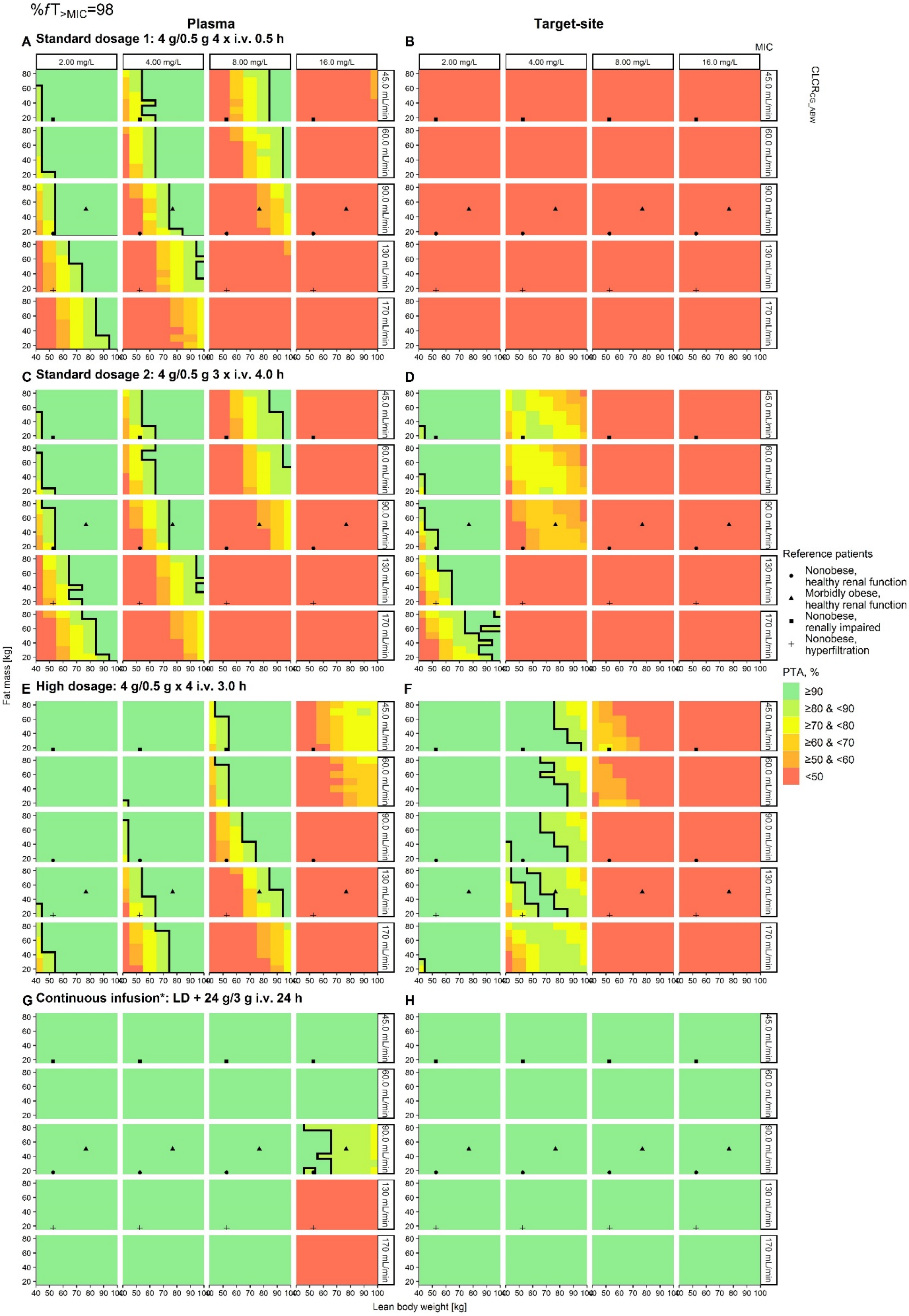


**Figure S14.** Probability of target attainment (PTA) based on the PK/PD target %$fT_{>MIC}$=98 versus lean body weight and fat mass in plasma (left panel) and target-site (right panel) stratified by (i) glomerular filtration rate according to CKD stages (moderate: 45.0–59.0 mL/min, mild: 60.0–98 mL/min, normal: >90 mL/min) or glomerular hyperfiltration (≥130 mL/min) covered by this study and (ii) MIC (2.00, 4.00, 8.00, 16.0 mg/L) after four different i.v. infusion regimens.

Asterisk: Pharmacokinetic/pharmacodynamic targets related to 4×MIC in plasma and 1xMIC at target-site; Reference patients: See description Table 1 in main manuscript. Bold black line: separates PTA≥90% (adequate therapy) from PTA<90%.

Abbreviations: $\%fT_{>MIC}$: Fraction of time that unbound concentration exceeds MIC over 24 h; CKD: Chronic kidney disease; $CLCR_{CG\_ABW}$: Creatinine clearance calculated via Cockcroft-Gault equation based on adjusted body weight; i.v.: Intravenous; LD: 4 g i.v. loading dose over 0.5 h; MIC: Minimum inhibitory concentration.

**References**


[1] Mould DR, Upton RN. Basic Concepts in Population Modeling, Simulation, and Model-Based Drug Development—Part 2: Introduction to Pharmacokinetic Modeling Methods. CPT Pharmacometrics Syst Pharmacol 2013;2. doi:10.1038/psp.2013.14.

[2] Hutmacher MM, Kowalski KG. Covariate selection in pharmacometric analyses: a review of methods. Br J Clin Pharmacol 2015;79:132–47. doi:https://doi.org/10.1111/bcp.12451.

[3] Dosne A-G, Bergstrand M, Karlsson MO. An automated sampling importance resampling procedure for estimating parameter uncertainty. J Pharmacokinet Pharmacodyn 2017;44:509–20. doi:10.1007/s10928-017-9542-0.

[4] Janmahasatian S, Duffull SB, Ash S, Ward LC, Byrne NM, Green B. Quantification of lean bodyweight. Clin Pharmacokinet 2005;44:1051–65.
